# Positive emotional contagion by laughter-like 50-kHz ultrasonic vocalizations in rats

**DOI:** 10.1016/j.isci.2026.118087

**Published:** 2026-11-02

**Authors:** Emilie Bartsoen, Robin Vloeberghs, Parnian Saberi, Kobe Desender, Markus Wöhr

**Affiliations:** 1 KU Leuven, Faculty of Psychology and Educational Sciences, Research Unit Brain and Cognition, Laboratory of Biological Psychology, Social and Affective Neuroscience Research Group, 3000 Leuven, Belgium; 2 KU Leuven, Leuven Brain Institute, 3000 Leuven, Belgium; 3 Philipps-University of Marburg, Faculty of Psychology, Experimental and Biological Psychology, Social and Affective Neuroscience, 35032 Marburg, Germany; 4 Philipps-University of Marburg, Center for Mind, Brain and Behavior, 35032 Marburg, Germany

**Keywords:** positive emotional contagion, empathy, affective resonance, acoustic startle response, ultrasonic vocalizations

## Abstract

Rats display advanced empathic abilities, yet current research is focused on empathy toward negative affect, largely neglecting positive affective states. We therefore developed a paradigm suitable for assessing both negative and positive emotional contagion. It relies on ethotransmission (i.e., acoustic emotional communication) of affective states to conspecifics via ultrasonic vocalizations (USV), i.e., alarming 22-kHz USV (negative affect) and laughter-like 50-kHz USV (positive affect). Specifically, we subjected rats to playback of 22-kHz or 50-kHz USV, while assessing their acoustic startle response (ASR), which is known to be emotionally modulated (i.e., potentiated and attenuated by negative and positive affect, respectively). Here, 50-kHz but not 22-kHz USV strongly attenuated the ASR across sexes and development, suggesting that laughter-like 50-kHz USV induced positive affect in receivers, indicative of positive emotional contagion. Our paradigm provides the first quantitative assessment of positive emotional contagion in rats through capitalizing on ethological signals (USV) and simple reflexes (ASR).

## Highlights

• 50-kHz ultrasonic vocalizations (USV) reflect a positive affective state in rats
• 50-kHz USV playback attenuates the acoustic startle response (ASR) in rats
• ASR attenuation is most prominent in rats emitting 50-kHz response calls
• ASR attenuation indicates positive emotional contagion across ages and sexes

## Graphical abstract

**Figure:**
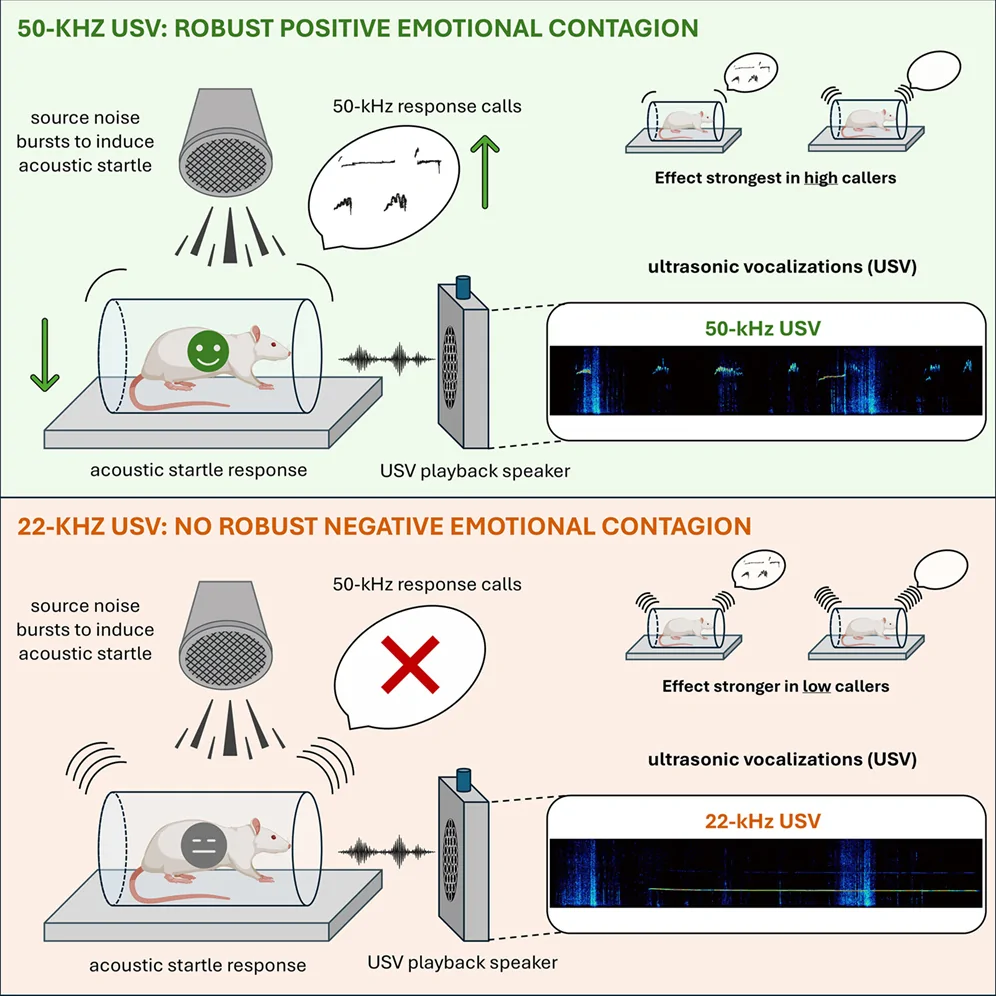

## Teaser

Acoustics; Behavioral neuroscience; Sensory neuroscience

## Introduction

The shared feeling of sadness when you see someone crying or of joy when someone else wins a gold medal can be called “emotional contagion”. Emotional contagion is the matching of affective states between two beings transmitted via certain modalities (e.g., auditory, visual, etc.)—something that happens automatically and does not require conscious awareness.^1^ This basic form of affective resonance is proposed to lie at the core of more complex forms of empathy (e.g., compassion) and pro-social behavior (e.g., helping behavior).^2^ Emotional contagion has been observed in several species and occurs in response to negative (e.g., fear) and positive (e.g., happiness) affective states.^3^^,^^4^^,^^5^^,^^6^^,^^7^

Rats are highly social and are equipped with a diverse set of affective (and cognitive) abilities, such as emotional contagion and other forms of empathy-like behavior (e.g., cooperation, helping behavior).^6^^,^^7^^,^^8^^,^^9^^,^^10^^,^^11^^,^^12^ Previous emotional contagion paradigms, however, focused primarily on negative affective states,^13^^,^^14^^,^^15^ leaving the positive equivalent neglected. To fully understand this phenomenon and the underlying neural systems, new paradigms to measure positive emotional contagion in rats are needed.

To communicate their affective state to conspecifics, numerous social species use acoustic emotional communication or “ethotransmission,” by which they generate negative or positive arousal in the receiver.^16^^,^^17^ For example, rats emit ultrasonic vocalizations (USV) to communicate their affective state to conspecifics.^18^ Juvenile and adult rats emit two main types of USV. 50-kHz USV are emitted in appetitive situations, such as during rough-and-tumble play^19^^,^^20^ or under the influence of psychostimulant drugs, most notably d-amphetamine.^21^ It is therefore assumed that these calls reflect a positive affective state, and were previously referred to as “rat laughter” and evolutionary “antecedents of human joy”.^22^ Through playback experiments (Figure 1A), it was found that these calls induce strong approach behavior,^24^ together with phasic dopamine release in the nucleus accumbens of the receiver.^25^ In contrast to the laughter-like 50-kHz USV, rats emit 22-kHz USV during aversive situations (e.g., detecting predator odors). They appear to function as alarm calls,^26^ and in line with this, rats display behavioral inhibition^24^^,^^27^ and amygdala activation upon hearing them.^28^ It appears likely that such overt behavioral changes, i.e., approach behavior versus behavioral inhibition, in the receiver are associated with respective alterations of its affective state.

**Figure 1:**
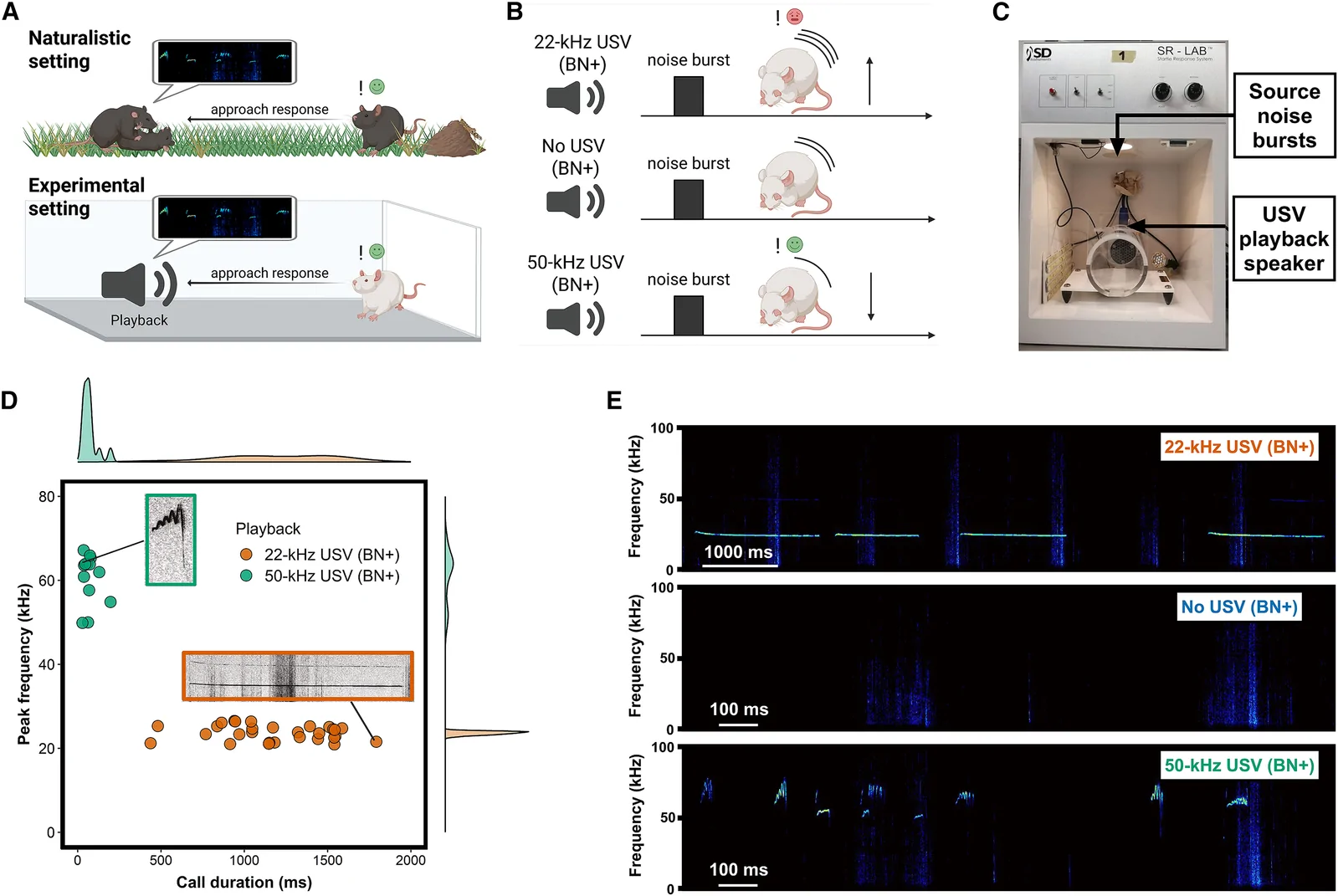
The emotional contagion paradigm: affective state transmission via USV in the ASR test (A) Graphical representation of emotional transmission via USV in a naturalistic versus experimental setting. In nature, emotional contagion occurs in response to a second party, such as witnessing playing rats while emitting 50-kHz USV that may provoke an approach response. However, in laboratory settings the transmitter of USV is often replaced by a loudspeaker to gain additional experimental control with high specificity (e.g., acoustic features) and to obtain a clear focus on the recipient’s behavioral response.^23^ (B) Hypotheses underlying the emotional contagion paradigm. Playback of 22-kHz USV and 50-kHz USV is expected to induce a negative and positive affective state, respectively. Successful affective state matching can in turn be measured by a potentiated (by 22-kHz USV) and attenuated (by 50-kHz USV) magnitude of the ASR. (C) Picture of the experimental setup. The startle response system was equipped with two loudspeakers, one for presenting noise bursts to evoke the ASR and one for USV playback. (D) Scatterplot outlining the distinct nature of 22-kHz USV and 50-kHz USV that were presented, in terms of duration (ms) and peak frequency (kHz). The kernel density estimate for each USV type on both characteristics is plotted on the side. (E) Spectrograms depicting acoustic playback stimuli presented during the ASR test of the first experiment. Please note the difference in time scaling, i.e., the scale bar represents 1000 ms in the spectrogram depicting 22-kHz USV (BN+) and 100 ms in the spectrograms depicting No USV (BN+) and 50-kHz USV (BN+).

A reliable readout of an organism’s affective state is the magnitude of the acoustic startle response (ASR). The ASR is a reflexive contraction of body muscles in response to a loud and sudden noise burst. It is evolutionarily conserved and can be observed in any organism from an early age that processes auditory signals.^29^ Interestingly, the magnitude of the ASR depends on the affective state of the organism. While an increased ASR is indicative of a negative affective state, a decreased ASR is indicative of a positive affective state. For example, classical conditioning studies in rats have repeatedly shown that a negative or positive affective state (induced by the presentation of an aversive or appetitive conditioned stimulus) enhances or attenuates the ASR, respectively, known as “fear-potentiated startle”^30^^,^^31^ and “pleasure-attenuated startle.”^31^^,^^32^ Emotional modulation of the ASR gives us therefore a window into the affective state of an organism, including that of rats.

To test the hypothesis that a sender can evoke changes in the affective state of receiving rats through emitting 22-kHz USV or 50-kHz USV, we developed a bidirectional (positive and negative) emotional contagion paradigm that expands on USV as ethotransmitters and the emotional modulation of the ASR. The emotional contagion paradigm consists of playback of 22-kHz USV, 50-kHz USV, and acoustic controls, presented during the ASR test (Figures 1B–1E). Both male and female rats were tested at juvenile and young adult ages (longitudinal design). In line with emotional contagion, we expected playback of 22-kHz USV and 50-kHz USV to induce a negative and positive affective state in the recipient rat, respectively. The extent to which the emotional state is transmitted via USV playback can in turn be measured by the level of ASR potentiation (by 22-kHz USV) and attenuation (by 50-kHz USV) compared to the acoustic control stimulus.

In line with our expectations, we found a strong ASR attenuation by 50-kHz USV playback in all age and sex groups. This effect was consistently observed across two experiments employing independent cohorts of rats, distinct experimental protocols, and different acoustic stimuli, indicative of robust positive emotional contagion. Interestingly, stable trait-like inter-individual differences were seen across development, and positive emotional contagion was particularly prominent in rats emitting 50-kHz response calls during 50-kHz USV playback. Playback of 22-kHz USV did not lead to robust negative emotional contagion leaving the ASR magnitude virtually unaltered in most rats.

## Results

### Playback of laughter-like 50-kHz USV attenuates acoustic startle reactivity

Playback of laughter-like 50-kHz USV exerted prominent effects on the ASR. To assess emotional contagion in the first experiment, male and female rats were subjected to the ASR test while hearing playback of 22-kHz USV (plus background noise, BN+), no USV (plus background noise, BN+), and 50-kHz USV (plus background noise, BN+) (Figure 2A). This was assessed longitudinally at juvenile and young adult age. Across noise burst intensities, 50-kHz USV (BN+) playback reduced the ASR, as compared to 22-kHz USV (BN+) and no USV (BN+). This effect was found in all age and sex groups, i.e., juvenile males (*χ*^2^[2] = 15.73, *p* = 0.0004), juvenile females (*χ*^2^[2] = 26.67, *p* <0.0001), adult males (*χ*^2^[2] = 52.97, *p* <0.0001), and adult females (*χ*^2^[2] = 20.61, *p* <0.0001). In contrast, 22-kHz USV (BN+) had no prominent impact on the ASR (Figures 2B–2E for *post-hoc* results).

**Figure 2:**
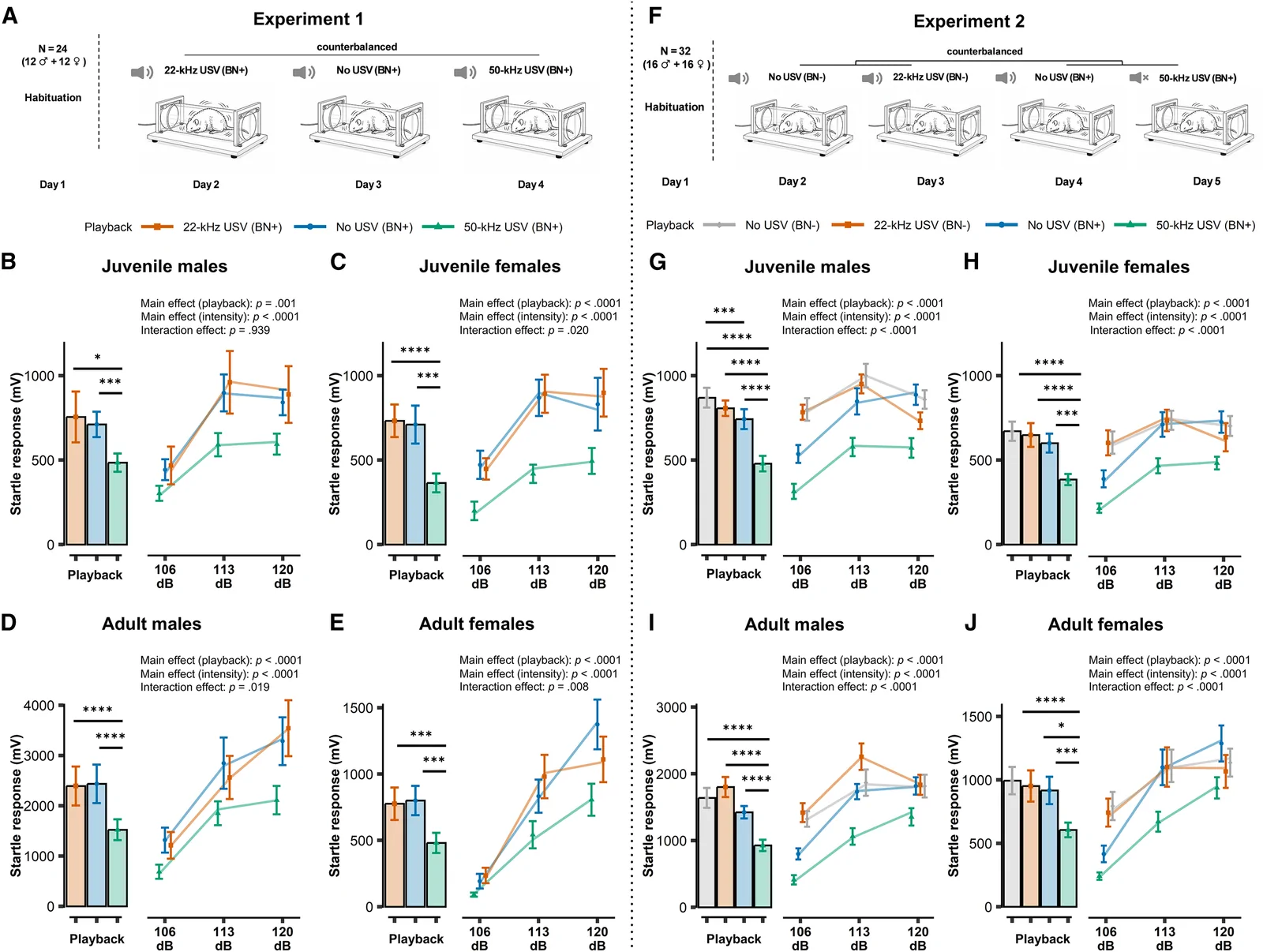
Positive emotional contagion: emotional modulation of the ASR via USV playback (A) In experiment 1, emotional contagion was assessed using playback of 22-kHz USV (BN+), no USV (BN+), or 50-kHz USV (BN+). The paradigm was conducted in (B) juvenile males (*N* = 12), (C) juvenile females (*N* = 12), (D) adult males (*N* = 12), and (E) adult females (*N* = 12). Each rat was tested at both ages in a within-subject design. Across all age and sex groups, playback of 50-kHz USV (BN+) significantly decreased the ASR virtually at all noise burst intensities compared to both no USV (BN+) and 22-kHz USV (BN+), indicating transmission of a positive affective state. (F) To assess the robustness of this effect, experiment 1 was repeated in an independent cohort of rats using a modified emotional contagion paradigm. Rats were again tested in a repeated within-subject test design as (G) juvenile males (*N* = 16), (H) juvenile females (*N* = 16), (I) adult males (*N* = 16), and (J) adult females (*N* = 16). Consistent with experiment 1, playback of 50-kHz USV (BN+) strongly attenuated the ASR compared to all other acoustic conditions across all age and sex groups. Together, these findings demonstrate robust positive emotional contagion mediated by laughter-like 50-kHz USV. Bar plots show the ASR to each playback condition across noise burst intensities (mean ± SE). Line graphs show the ASR for each playback condition separated by noise burst intensity, including observed data (mean ± SE) and model predictions (mean [lines]). ∗*p* <0.05, ∗∗*p* <0.01, ∗∗∗*p* <0.001, ∗∗∗∗*p* <0.0001.

Noise burst intensity-specific analyses revealed a clear ASR modulation depending on the acoustic stimulus presented in all age and sex groups (main effect playback: *χ*^2^[2] = 13.53, *p* = 0.001 [juvenile males], *χ*^2^[2] = 28.96, *p* <0.0001 [juvenile females], *χ*^2^[2] = 28.10, *p* <0.0001 [adult males], *χ*^2^[2] = 20.63, *p* <0.0001 [adult females]). As expected, the ASR magnitude increased with increasing noise burst intensity (main effect intensity: *χ*^2^[2] = 107.63, *p* <0.0001 [juvenile males], *χ*^2^[2] = 140.08, *p* <0.0001 [juvenile females], *χ*^2^[2] = 228.96, *p* <0.0001 [adult males], *χ*^2^[2] = 193.69, *p* <0.0001 [adult females]), but the level of increment differed substantially between playback conditions in most groups (interaction effect: *χ*^2^[4] = 0.80, *p* = 0.939 [juvenile males], *χ*^2^[4] = 11.72, *p* = 0.020 [juvenile females], *χ*^2^[4] = 11.82, *p* = 0.019 [adult males], *χ*^2^[4] = 13.68, *p* = 0.008 [adult females]). Specifically, 50-kHz USV (BN+) induced a marked attenuation of the ASR compared to both no USV (BN+) and 22-kHz USV (BN+), although the strength of this effect varied across groups depending on noise burst intensity. Finally, 22-kHz USV (BN+) did not potentiate the ASR, as its magnitude did not differ from no USV (BN+) (Figure S2 for *post-hoc* results).

In sum, experiment 1 showed a strong level of ASR attenuation by 50-kHz USV (BN+) playback, which suggests a successful transmission of the positive affective state of the sender—via 50-kHz USV—to the receiver (i.e., positive emotional contagion). Playback of 22-kHz USV (BN+) did not change the ASR magnitude, suggesting that they did not induce a negative affective state.

Because 22-kHz USV naturally function as alarm calls that signal potential threat,^17^ the added background noise (BN+) may have weakened their biological salience. To test this and to assess the robustness of the 50-kHz USV effect, the experiment was repeated using a modified emotional contagion protocol with a different set of control conditions in an independent cohort of rats. To this end, juvenile and young adult male and female rats were subjected to the ASR test during playback of four conditions: no USV (BN-), 22-kHz USV (BN-), no USV (BN+), and 50-kHz USV (BN+) (Figure 2F).

The result pattern of experiment 1 was replicated. 50-kHz USV (BN+) playback strongly reduced the ASR in all age and sex groups. Across noise burst intensities, playback of 50-kHz USV (BN+) reduced the ASR, as compared to all the other acoustic playback conditions. Again, this was found in all age and sex groups, i.e., juvenile males (*χ*^2^[2] = 301.57, *p* <0.0001), juvenile females (*χ*^2^[2] = 34.75, *p* <0.0001), adult males (*χ*^2^[2] = 67.21, *p* <0.0001), and adult females (*χ*^2^[2] = 30.23, *p* <0.0001) (Figures 2G–2J for *post-hoc* results). Of note, no USV (BN+) playback modestly reduced the ASR, as compared to 22-kHz USV (BN-) and no USV (BN-), particularly in juvenile males, yet by far not as apparent as the effect induced by 50-kHz USV (BN+) playback. This indicates that background noise (BN+) alone cannot account for the robust ASR suppression across noise burst intensities during 50-kHz USV presentation. Similar to 22-kHz USV (BN+) in experiment 1, 22-kHz USV (BN-) did not potentiate the ASR, suggesting that the added background noise (BN+) was not the primary factor reducing the behavioral impact of 22-kHz USV. Of note, noise burst intensity-specific results for experiment 2 are provided in the Supplementary Materials (Text S1; corresponding data and *post-hoc* comparisons are shown in Figures 2G–2J and S3, respectively).

Taken together, playback of 50-kHz USV led to a strong attenuation of the ASR. In contrast to the other acoustic stimuli, 50-kHz USV attenuated the ASR virtually across all noise burst intensities. This effect was seen in both male and female rats and occurred irrespective of developmental stage, i.e., at juvenility and adulthood. Importantly, the effect was reproduced in two experiments testing two independent cohorts of rats in two slightly different versions of the paradigm, highlighting its robustness. This strongly supports the notion that positive emotional contagion induced by 50-kHz USV is a stable phenomenon. In contrast, 22-kHz USV playback did not consistently modulate the ASR in either experiment, indicating that these calls were insufficient to induce a robust negative emotional contagion response under our experimental conditions.

### Playback of laughter-like 50-kHz USV elicits response calls

To obtain a second independent readout of emotional contagion, a detailed spectrographic analysis of the USV emitted by the juvenile rats exposed to the emotional contagion paradigm (experiment 1), i.e., response calls,^33^ was performed. In general, USV emission was abundant during the test with 91.67% (22/24) rats vocalizing at least once on each playback day, resulting in a total of *N* = 1247 calls. The majority of these calls were typical 50-kHz USV (*N* = 1205, 96.63%), followed by atypical 22-kHz USV (*N* = 25, 2.00%), atypical 50-kHz USV (*N* = 12, 0.96%), and typical 22-kHz USV (*N* = 5, 0.40%) (Figures 3A and 3B). Playback of 50-kHz USV (BN+) elicited the strongest and most diverse vocal repertoire, as less common call types occurred proportionally more frequent during these test sessions, although typical 50-kHz USV still accounted for the vast majority of emitted calls (Table S2). As most emitted calls consisted of typical 50-kHz USV, subsequent analyses focused on this call category.

**Figure 3:**
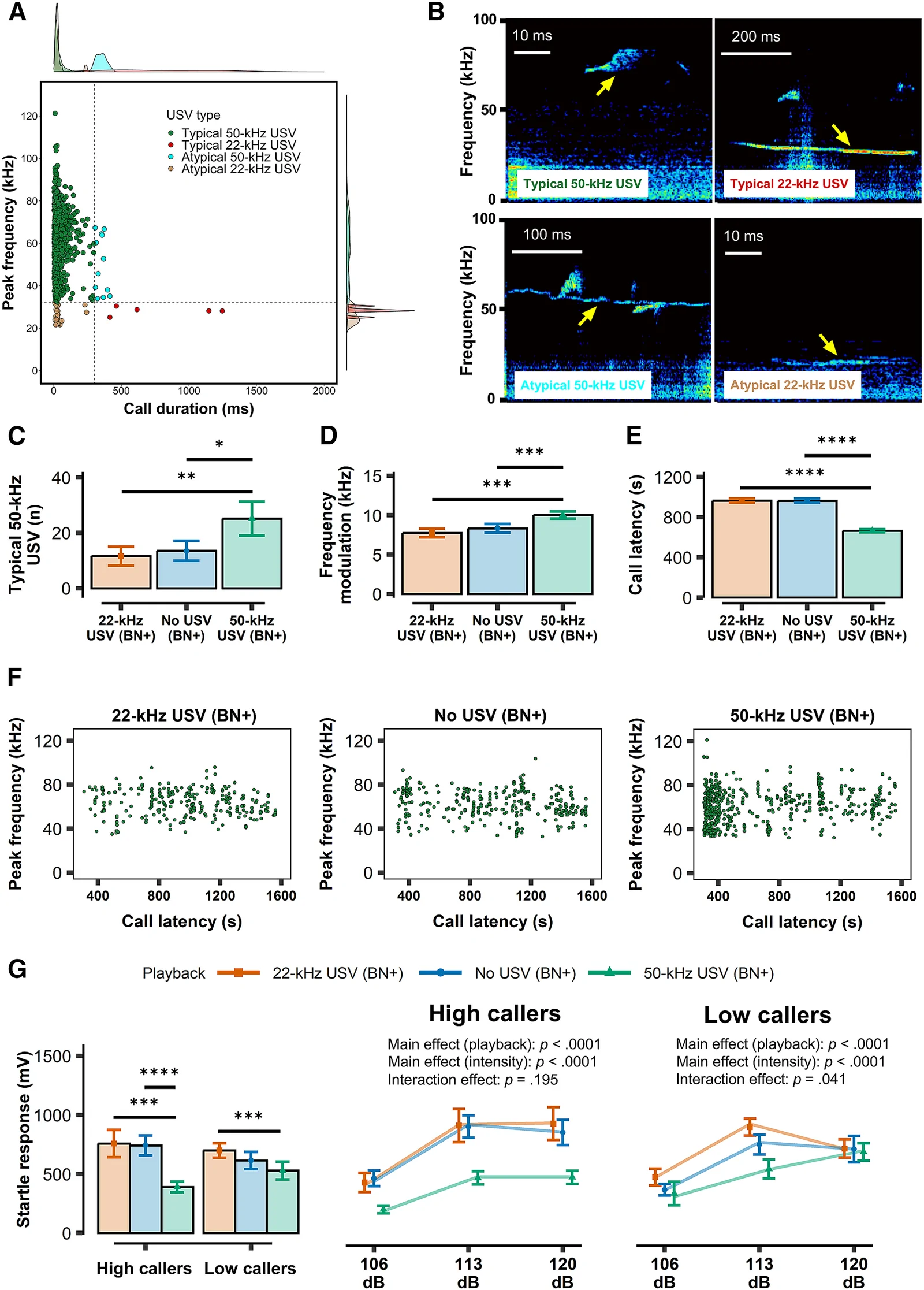
Positive emotional contagion: emotional modulation of the emission of response calls via USV playback (A) Scatterplot depicting all USV (*N* = 1247 calls from *N* = 24 rats at juvenile age) emitted during the emotional contagion paradigm, in terms of peak frequency (kHz) and duration (ms), subdivided in four USV types. Dashed lines represent the cut-off values on peak frequency (kHz) and duration (ms) that distinguishes the subtypes. The kernel density estimate for each USV type on both characteristics is plotted on the side. (B) An example observed of each USV type. Please note the difference in time scaling, i.e., the scale bar represents 10 ms in the spectrogram depicting typical 50-kHz USV, 200 ms in the spectrogram depicting typical 22-kHz USV, 100 ms in the spectrogram depicting atypical 50-kHz USV, and 10 ms in the spectrogram depicting atypical 22-kHz USV. (C) Bar plot (mean ± SE) comparing the number of typical 50-kHz USV observed during each playback condition per rat (analyzed on rat level, *N* = 24). (D and E) Bar plots (mean ± SE) comparing frequency modulation (kHz) and average call latency (s), respectively, of typical 50-kHz USV (analyzed on call level, *N* = 1205) emitted in each playback condition. (F) A time course is plotted of the call latency (i.e., when the call was emitted) during the test session. A separate plot for each playback condition is depicted. Calls starting from 300 s were considered, since this is when playback was initiated. (G) Comparing the effect of ASR modulation by playback subdividing the rats into high callers (*N* = 18) and low callers (*N* = 6). Bar plots show the ASR to each playback condition across noise burst intensities (mean ± SE). Line graphs show the ASR for each playback condition separated by noise burst intensity, including observed data (mean ± SE) and model predictions (mean [lines]). ∗*p* <0.05, ∗∗*p* <0.01, ∗∗∗*p* <0.001, ∗∗∗∗*p* <0.0001.

The number of typical 50-kHz USV differed between playback conditions (*χ*^2^[2] = 12.31, *p* = 0.002; Figure 3C), and were more frequently emitted during 50-kHz USV (BN+) playback, as compared to 22-kHz USV (BN+) (*p* = 0.002) and no USV (BN+) playback (*p* = 0.042). Not only did they occur more frequently during 50-kHz USV (BN+) playback, but the typical 50-kHz USV emitted were characterized by a higher frequency modulation (*H*[2] = 25.62, *p* <0.0001; Figure 3D). Call duration (*H*[2] = 1.05, *p* = 0.591) and peak frequency (*F*[2] = 2.79, *p* = 0.062) of the typical 50-kHz USV did not differ between playback conditions (Figure S3). Interestingly, analysis of call latency across the test session revealed that rats vocalized most strongly at the onset of 50-kHz USV (BN+) playback, after which calling rate rapidly declined (Figure 3F), whereas call latency appeared more random during the other two playback conditions. This contrast in temporal pattern suggests that 50-kHz USV (BN+) specifically elicits 50-kHz USV emission, rather than sustained spontaneous calling. This is further supported by a lower average call latency during 50-kHz USV (BN+) playback (*H*[2] = 191.44, *p* <0 .0001; Figure 3E), as compared to both 22-kHz USV (BN+) (*p* <0.0001) and no USV (BN+) playback (*p* <0.0001).

In sum, our analyses showed that 50-kHz USV (BN+) playback elicited response calls in the 50-kHz USV range that were strongly frequency modulated. Importantly, 50-kHz USV, in particular the frequency-modulated ones, are widely considered indicators of positive affect.^34^ Together, these findings provide independent evidence that playback of 50-kHz USV induced positive emotional contagion beyond the effects observed in the ASR.

### High callers display stronger startle reactivity attenuation during 50-kHz USV playback

Next, we assessed whether ASR attenuation depends on inter-individual differences in the emission of 50-kHz response calls. To achieve this, rats were subdivided into high callers (>4 typical 50-kHz USV; *N* = 18) emitting 50-kHz USV in response to 50-kHz USV playback and low callers (≤4 typical 50-kHz USV; *N* = 6), based on their individual call profile. Playback had profoundly different effects on ASR modulation between both groups (*χ*^2^[2] = 33.74, *p* <0.0001 [high callers], *χ*^2^[2] = 15.13, *p* <0.001 [low callers]). Across noise burst intensities, 50-kHz USV (BN+) playback produced a pronounced ASR attenuation in high callers compared to the other playback conditions. In contrast, low callers displayed less attenuation. Instead, a modest potentiation of startle reactivity by 22-kHz USV (BN+) playback was observed relative to 50-kHz USV (BN+) but not to no USV (BN+) (Figure 3G).

An in-depth analysis of the different noise burst intensities confirmed that high callers displayed a robust reduction in ASR magnitude by 50-kHz USV (BN+) (main effect playback: *χ*^2^[2] = 31.99, *p* <0.0001), independent of which noise burst intensity was presented (interaction effect: *χ*^2^[2] = 6.05, *p* = 0.195; Figure 3G, *post-hoc* analyses Figure S5). In contrast, although the ASR in low callers was also modulated by playback (main effect playback: *χ*^2^[2] = 19.91, *p* <0.0001), modulation only became visible depending on noise burst intensity (interaction effect: *χ*^2^[2] = 9.99, *p* = 0.041). More specifically, low callers only displayed prominent ASR attenuation at the intermediate noise burst intensity (i.e., 113 dB). Interestingly, this modest effect appeared to be partly driven by a slight potentiation of the ASR during 22-kHz USV (BN+) playback (Figure S5).

Together, these findings indicate that the modulation of ASR by USV playback is linked to the rat’s own vocal behavior. Rats that actively emitted response calls in the 50-kHz USV range, i.e., the ones displaying a particularly high level of positive affect, showed a pronounced attenuation of ASR during 50-kHz USV (BN+) playback, whereas low calling rats, i.e., the ones not displaying a particularly high level of positive affect, were comparatively insensitive to playback of 50-kHz USV and showed greater responsiveness to 22-kHz USV. This further supports the notion that that the ASR attenuation in response to 50-kHz USV playback is a reliable measure of positive emotional contagion in rats. Since our findings predominantly indicate an effect of 50-kHz USV but not 22-kHz USV playback both on the level of ASR and the emission of response calls, our subsequent analyses on age and sex differences solely focused on the effects of 50-kHz USV (BN+) playback compared to no USV (BN+). To this aim, the data of experiment 1 and 2 were pooled because their results were highly similar.

### Positive emotional contagion is stable across sexes and development

To assess age and sex differences on positive emotional contagion, the ASR was transformed to percentage startle modulation by 50-kHz USV (BN+) compared with no USV (BN+) to account for weight differences.^35^^,^^36^ This value was compared between (1) juvenile males and females (Figure 4A), (2) adult males and females (Figure 4B), (3) juvenile and adult males (Figure 4C), and (4) juvenile and adult females (Figure 4D). In line with previous analyses, playback of 50-kHz USV (BN+) consistently attenuated the ASR, as compared to no USV (BN+). This was indicated by a negative percentage startle modulation in both sexes across development both at the pooled and at the individual noise burst intensity level. For example, the percentage startle modulation reached −89.43% for a specific juvenile female rat, meaning that upon hearing the laughter-like 50-kHz USV, her startle response magnitude decreased by 89.43% compared to no USV (BN+). The robustness of this phenomenon also came forward when comparing the age and sex groups directly, showing no differences between groups, in the pooled and noise burst intensity-specific analyses (Figures 4A–4D; all *p* >0.117). In conclusion, playback of 50-kHz USV evoked positive emotional contagion similarly in both sexes and is stable throughout development.

**Figure 4:**
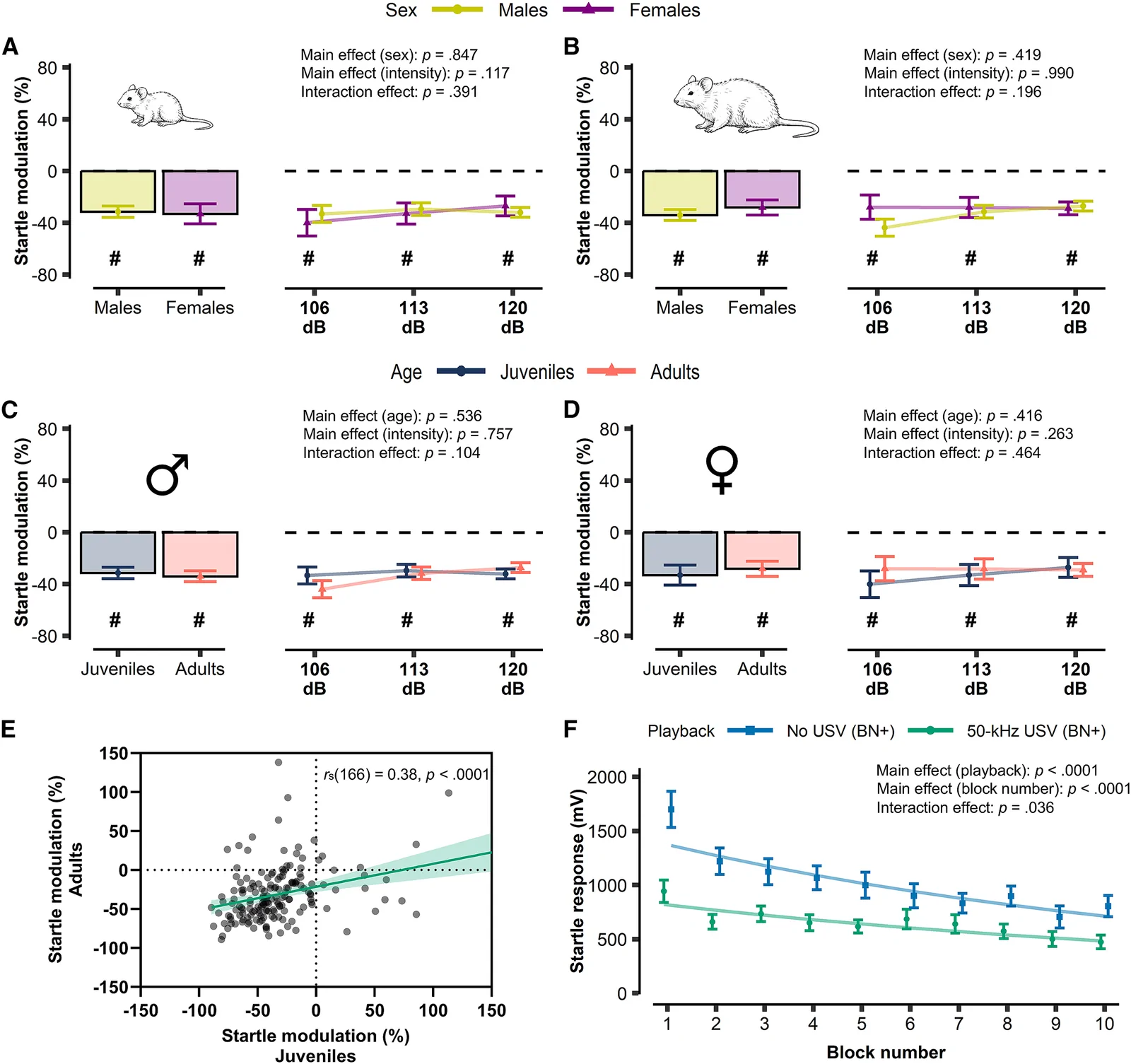
Positive emotional contagion: sex, age, and trait-like inter-individual differences in the emotional modulation of the ASR via USV playback Sex effects in (A) juveniles (*N* = 56) and (B) adults (*N* = 56) are presented, as well as age effects in (C) males (*N* = 56) and (D) females (*N* = 56). Bar plots represent the observed (mean (point) ± SE (bar)) percentage startle modulation by 50-kHz USV (BN+) playback compared with no USV (BN+) across noise burst intensities. Line graphs show the percentage startle modulation separated by noise burst intensity, including observed data (mean ± SE) and model predictions (mean [lines]). Data from experiment 1 and 2 were pooled since the ASR was strongly attenuated by 50-kHz USV (BN+) playback in both experiments, further indicated by the negative percentage startle modulation for all age and sex groups at all noise burst intensities. No differences between sexes and age groups were detected, indicating the stability of this response. (E) Scatterplot depicting the correlation between percentage startle modulation by 50-kHz USV (BN+) playback as juveniles (*N* = 56) (on *x* axis) and adults (*N* = 56) (on *y* axis). Data of males and females were pooled since no sex effects were detected earlier. Each dot represents a rat with a calculated percentage modulation per noise burst intensity (*N* = 168; black dots indicate superimposed rats for clarity against the gray background). A simple linear regression line ±SE, predicting percentage startle modulation by age, is plotted. (F) Temporal pattern of startle response modulation by 50-kHz USV (BN+) playback versus no USV (BN+) playback across blocks of presentation. Data of juvenile and adult males and females were pooled (*N* = 112), since no differences were detected. Graphs depict model predictions (mean [lines]) and observed data (mean [point] ±SE [bar]). The lines gradually move closer together, reflecting a decrease in startle modulation by 50-kHz USV playback over blocks. #*p* <0.01 50-kHz USV (BN+) startle attenuation compared to no USV (BN+).

### Positive emotional contagion is characterized by stable intra-individual differences

To test whether positive emotional contagion is characterized by stable intra-individual differences across development, the percentage startle modulation by 50-kHz USV (BN+) relative to no USV (BN+) was calculated for each rat at juvenility and adulthood to correct for weight differences.^35^^,^^36^ Male and female data were pooled, since no sex differences were found in our previous analyses. A positive correlation was found between juvenile and adult ages for the percentage startle modulation by 50-kHz USV (BN+) playback (*r*_*s*_*[166]* = 0.38, *p* <0 .0001; Figure 4E). More specifically, this means that rats with a strong percentage startle attenuation by 50-kHz USV (BN+) at juvenile age also showed a strong percentage startle attenuation during adulthood, and vice versa. Our results suggest that positive emotional contagion is indeed characterized by stable intra-individual differences throughout the life course, suggesting a trait-like phenotype.

### Startle reactivity attenuation by laughter-like 50-kHz USV decreases over time

As opposed to a chronic state or mood, emotional contagion is a short-lived phenomenon driven by the social transmission of discrete emotional states from the sender to the receiver, elicited by a specific stimulus.^37^ In line with this, we found that playback of 50-kHz USV induced the strongest startle reactivity attenuation during the first blocks of presentation which slowly decreased during the second half of the test (interaction effect: *χ*^2^[1] = 4.37, *p* = 0.036; Figure 4F). These results further support that the ASR attenuation by 50-kHz USV (BN+) playback reflects an emotional state driven process.

## Discussion

The present study investigated whether USV can induce emotional contagion in rats using a novel ASR based paradigm. Two experiments were conducted employing independent cohorts of male and female rats tested both as juveniles and adults. In the first experiment, rats were exposed to 50-kHz USV (appetitive social contact calls), 22-kHz USV (aversive social alarm calls), and a no-USV control condition, all presented against background noise (BN+) to maximize acoustic comparability. In both sexes, 50-kHz USV (BN+) consistently attenuated startle reactivity across ages, whereas 22-kHz USV (BN+) produced little or no effect in most rats. Because 22-kHz USV function as alarm signals that induce vigilance and defensive behavior,^17^ it remained possible that the added background noise (BN+) reduced their biological salience. Therefore, a second experiment was conducted in which 22-kHz USV and an additional no-USV control condition were used without background noise (BN−), while the 50-kHz USV (BN+) and no-USV (BN+) conditions were retained. This experiment replicated the marked ASR attenuation by 50-kHz USV (BN+) but again did not reveal a clear ASR potentiation during 22-kHz USV (BN−) playback. As in the first experiment, the ASR attenuation by 50-kHz USV (BN+) playback was highly robust, occurring in males and females during both juvenility and adulthood, reflecting stable individual differences across developmental stages. Together, the consistent findings obtained across two independent cohorts of rats indicate that 50-kHz USV playback reliably induces a positive affective state in receiving rats and provide strong support for the hypothesis that these calls can evoke positive emotional contagion.

Positive emotional contagion was assessed through the emotional modulation of the ASR^38^ and playback of 50-kHz USV, which serves as a strong ethotransmitter of positive affect.^16^ The emotional modulation of the ASR is a refined method for assessing affective state changes in both animals and humans.^38^ Its working mechanism is based on the motivational priming hypothesis,^39^ which suggests that emotional states activate corresponding neural systems, prioritizing congruent actions while inhibiting incongruent ones. As part of a defensive response, the ASR increases with aversive affective states and decreases with appetitive affective states.^39^ This bidirectional pattern has been validated across species, and is now better known as the fear-potentiated and pleasure-attenuated startle.^30^^,^^31^^,^^32^

In our study, the ASR of rats was strongly reduced by playback of 50-kHz USV in two separate experiments employing independent cohorts of rats. Moreover, ASR attenuation during 50-kHz (BN+) playback was accompanied by the emission of 50-kHz USV, i.e., response calls,^33^ by the receivers. 50-kHz USV, known as “rat laughter,”^22^ express the positive affective state of the transmitter and are emitted during appetitive situations (e.g., rough-and-tumble play).^19^^,^^20^^,^^40^ The receivers, in turn, find these calls highly rewarding, as shown by their approach behavior and instrumental generation of playback.^19^^,^^24^ In addition, hearing these calls activates the mesolimbic dopaminergic system, a brain circuit involved in reward processing.^25^ Therefore, similar to pleasure-attenuated startle, hearing 50-kHz USV likely triggered a positive affective state in these rats—potentially via dopamine signaling^25^—resulting in a reduced ASR. However, in contrast to previous studies,^30^^,^^32^ which relied on conditioning processes, the transmission of the affective state through 50-kHz USV here results from affective resonance between sender and receiver, making it a promising measure of positive emotional contagion.

An alternative explanation for the observed attenuation of the ASR, though not mutually exclusive with positive emotional contagion, is that 50-kHz USV playback induced an anxiolytic state. This interpretation is plausible, given that anxiolytic drugs have been shown to markedly reduce the ASR magnitude in rats.^41^^,^^42^ However, several aspects of our findings speak for the induction or sharing of a positive affective state as the primary driving force of the ASR reduction. Firstly, in parallel with the robust attenuation of the ASR, 50-kHz USV playback elicited strong vocal responses, i.e., response calls, particularly in the 50-kHz USV range.^33^ As 50-kHz USV are reliably evoked by appetitive stimuli, suppressed under aversive conditions, and are therefore widely regarded as indicators of positive affect in rats,^18^ this supports positive affective state induction. Secondly, the 50-kHz USV emitted during 50-kHz USV playback exhibited greater frequency modulation than those recorded in the other playback conditions. Frequency-modulated 50-kHz USV were suggested to be more closely associated with positive emotional arousal, whereas flat 50-kHz USV likely serve primarily social contact functions.^16^^,^^34^ The predominance of frequency-modulated response calls during 50-kHz USV playback therefore further supports the view that the rats were not merely less anxious, but were actively experiencing a positive affective state. Thirdly, consistent with this interpretation, ASR attenuation was most pronounced in rats emitting high rates of 50-kHz response calls and weaker in relatively silent animals. Furthermore, emotional contagion is expected to involve the transient matching of a conspecific’s affective state rather than a sustained shift in arousal.^37^ In line with this, the strongest ASR attenuation coincided with the highest rates of 50-kHz response calling during the initial playback blocks and gradually declined thereafter. This temporal profile closely resembles previous findings showing peak dopaminergic and behavioral responses when rats first encounter positive social signals, whether through 50-kHz USV playback or direct social interaction.^25^^,^^43^ Taken together, the convergence of behavioral, vocal, and temporal evidence is consistent with positive emotional contagion as the primary contributor to the observed reduction in the ASR.

Similar levels of positive emotional contagion were found in males and females, regardless of age, as indicated by comparable percentages startle attenuation by 50-kHz USV playback. While prior research suggests empathy-like behavior may be stronger in juveniles (e.g., approach behavior toward 50-kHz USV^24^) and females (e.g., helping behavior^10^), overt expressions of empathy are differently regulated than its emotional driver.^12^ In line with this, there is no unequivocal evidence for sex differences in negative emotional contagion in response to fear/pain in rats.^13^^,^^44^ To our knowledge, no studies have examined sex and age effects on positive emotional contagion in rats. However, due to its evolutionary benefits (e.g., promoting social bonding, stress resilience, resource detection),^14^^,^^15^ positive emotional contagion remains advantageous throughout life for both sexes, and although further research is needed, could potentially favor the evolution of stable levels as seen in our study.

Unlike the strong ASR attenuation by 50-kHz USV, 22-kHz USV playback had no effect yet on startle reactivity, suggesting these calls did not alter the receiver’s affective state. This was the case for both our experiments, including the second experiment, in which background noise was removed (22-kHz USV (BN–)) to mimic more naturalistic conditions (i.e., the emission of 22-kHz USV during freezing and therefore in absence of background noise). The lack of an apparent ASR alteration was unexpected, as a bidirectional pattern—startle potentiation (negative emotional contagion) and attenuation (positive emotional contagion)—was anticipated based on the social functions of these calls (alarm vs. laughter-like) and their emission contexts (aversive vs. appetitive).^18^^,^^26^ While rats emitting 22-kHz USV likely experience negative affect (e.g., display an enhanced startle response during emission^45^), results from playback studies appear less clear-cut.^46^ Similar to our findings, 50-kHz USV playback generally evoked strong behavioral responses in the receiver, whereas no or limited responses were observed by 22-kHz USV.^8^^,^^9^^,^^47^ More research is needed, but studies suggest that the contagiousness of 22-kHz USV depends on social context (e.g., proximity of conspecifics), previous experiences (e.g., autoconditioning), and other unknown factors.^17^^,^^46^ Distance to the source of danger and inter-individual differences in relevant traits, such as anxiety, likely play a role as well. Interestingly, 22-kHz USV playback produced a moderate increase in startle reactivity in rats that emitted few or no 50-kHz response calls. This finding raises the possibility that alarm calls are more ethologically salient to individuals with a more anxiety-prone phenotype.^48^ Although this interpretation requires direct testing, it suggests that susceptibility to negative emotional contagion via 22-kHz USV may depend on baseline anxiety levels. Future studies appear beneficial to investigate the relationship between trait anxiety and responsiveness to 22-kHz USV playback.

### Implications: A new behavioral assay for positive emotional contagion

Our study introduces a novel behavioral assay that reliably induces and quantifies positive emotional contagion in rats, addressing a crucial gap in empathy research, which has largely focused on negative affective states.^13^^,^^14^^,^^15^ While a few previous studies have successfully measured positive emotional contagion,^5^^,^^8^^,^^49^ they often require extensive training, prolonged testing periods/analyses, or rely on overt behaviors that may introduce confounds related to motivation or motor function. In contrast, our paradigm leverages ethologically relevant social signals (USV) and an automatic, reflexive readout (ASR). This eliminates the need for training and significantly reduces testing time to just four days. Moreover, it is minimally aversive, which improves animal welfare, and avoids the motor and motivational confounds via the ASR reflex.^50^ By offering a fast, reliable, and ethologically valid approach, our paradigm can pave the way for investigating links to other empathy-like behaviors, exploring neurobiological mechanisms, and examining changes in rodent models of neuropsychiatric disorders such as autism spectrum disorder.

### Limitations of the study

The current study has limitations that warrant further investigation. First, we did not monitor the estrous cycle in the adult female rats. Although estrous phase has not, to our knowledge, been directly linked to positive emotional contagion in rats, ovarian hormonal levels has been shown to influence socio-affective processing in rats, including negative emotional contagion and the production of 50-kHz USV, particularly in mating-related contexts.^51^^,^^52^ Consequently, we cannot exclude the possibility that estrous phase contributed to some of the variability observed in female responses. However, the fact that the reduction in startle reactivity was consistently seen in response to playback of 50-kHz USV in both sexes and at both developmental stages speaks against a major modulatory role of the estrous cycle. Second, here we aimed to establish a new paradigm using a 50-kHz USV playback file known to exert robust behavioral effects.^24^^,^^25^^,^^28^ It will be interesting to assess the generalizability of our findings across calls generated in different contexts. Finally, the present study focused primarily on behavioral outcomes and did not include neurobiological measures. As a result, the neural mechanisms underlying the observed attenuation of the ASR remain unknown. It will be interesting to assess whether the observed ASR attenuation by 50-kHz USV playback is accompanied by activation in relevant neural structures, such as the mesolimbic dopaminergic system.

## Resource availability

### Lead contact

Further information and requests for resources should be directed to and will be fulfilled by the lead contact, Markus Wöhr (markus.wohr@kuleuven.be).

### Materials availability

This study did not generate new unique reagents.

### Data and code availability

• Raw data generated by the SR-LAB-Startle Response System and SR-LAB Software (.accbd files), generated excel files used for data analysis along with a word document explaining all variables have been deposited at Open Science Framework and are publicly available as of the date of publication (https://doi.org/10.17605/OSF.IO/QRTZ3).
• All original code has been deposited at Open Science Framework and is publicly available as of the date of publication (https://doi.org/10.17605/OSF.IO/QRTZ3).
• Any additional information required to reanalyze the data reported in this study is available from the lead contact upon request.

## Acknowledgments

This study was supported by the 10.13039/501100003130Fonds voor Wetenschappelijk Onderzoek - Vlaanderen (FWO; Research Foundation – Flanders) through a PhD Fellowship to E.B. (11PCN24N) and a senior research project (G0C0522N) and an infrastructure project (I007022N) to M.W., the Deutsche Forschungsgemeinschaft (DFG, German Research Foundation) through a collaborative research center project (project-ID 422744262 – CRC 289; gefördert durch die Deutsche Forschungsgemeinschaft (DFG) – Projektnummer 422744262 – TRR 289) to M.W., and the Interne Fondsen KU Leuven (Internal Funds KU Leuven) through a BOFZAP Starting Grant (PXF-E0120-STG/20/062) to M.W.. The authors wish to thank Aisha Dondeyne and Samantha Piers for their assistance during data collection of the second experiment.

## Author contributions

Conceptualization, E.B. and M.W.; methodology, E.B. and M.W.; data collection, E.B. and P.S.; data analysis, E.B. and R.V., with help of K.D. and M.W.; writing – original draft, E.B. and M.W.; writing – review and editing, E.B., R.V., P.S., K.D., and M.W.; funding acquisition, E.B. and M.W.; supervision, K.D. and M.W.

## Declaration of interests

The authors declare no competing interests.

## Declaration of generative AI and AI-assisted technologies in the manuscript preparation process

During the preparation of this work, the authors used ChatGPT (GPT-5.6 Luna) for minor language editing purposes in order to improve readability and for the creation of Figure illustrations, more specifically the rat in the startle tube illustration used in Figures 2A and 2F, and the juvenile and adult rat illustrations used in Figures 4A and 4B. After using this tool or service, the authors reviewed and edited the content as needed and take full responsibility for the content of the publication.

## STAR★Methods

### Key resources table

**Table:** 

REAGENT or RESOURCE	SOURCE	IDENTIFIER
**Deposited data**		

Raw and analyzed data	This paper	https://doi.org/10.17605/OSF.IO/QRTZ3
Rstudio code for running the statistical analyses	This paper	https://doi.org/10.17605/OSF.IO/QRTZ3

**Experimental models: Organisms/strains**		

Male Sprague-Dawley Rat (Crl:CD(SD)) <Crl:CD(SD)>, age ∼24 days upon arrival	Charles River Laboratories, Saint-Germain-Nuelles, France	https://www.criver.com/
Female Sprague-Dawley Rat(Crl:CD(SD)) <Crl:CD(SD)>, age ∼24 days upon arrival	Charles River Laboratories, Saint-Germain-Nuelles, France	https://www.criver.com/

**Software and algorithms**		

RStudio	Posit Software, PBC, Boston, Massachusetts, USA	RRID: SCR_000432; https://posit.co/products/open-source/rstudio
BioRender	BioRender, Toronto, Ontario, Canada	RRID: SCR_018361; https://www.biorender.com/
GraphPad Prism	GraphPad Software, San Diego, California, USA	RRID: SCR_002798; https://www.graphpad.com/features
SR-LAB Software	San Diego Instruments, San Diego, California, USA	https://sandiegoinstruments.com/product/sr-lab-startle-response/
Avisoft SASLabPro	Avisoft Bioacoustics, Glienicke/Nordbahn, Germany	RRID:SCR_014438; https://avisoft.com/
Avisoft-RECORDER	Avisoft Bioacoustics, Glienicke/Nordbahn, Germany	RRID:SCR_014436; https://avisoft.com/
Raw and analyzed data	This paper	https://doi.org/10.17605/OSF.IO/QRTZ3
Rstudio code for running the statistical analyses	This paper	https://doi.org/10.17605/OSF.IO/QRTZ3

**Other**		

SR-LAB-Startle Response System	San Diego Instruments, San Diego, California, USA	https://sandiegoinstruments.com/product/sr-lab-startle-response/
SR-LAB Standardization Unit	San Diego Instruments, San Diego, California, USA	https://sandiegoinstruments.com/product/sr-lab-startle-response/
UltraSoundGate Player 116	Avisoft Bioacoustics, Glienicke/Nordbahn, Germany	https://avisoft.com/
Ultrasonic Dynamic Speaker Vifa	Avisoft Bioacoustics, Glienicke/Nordbahn, Germany	https://avisoft.com/
UltraSoundGate Condenser Microphone CM16	Avisoft Bioacoustics, Glienicke/Nordbahn, Germany	https://avisoft.com/
Avisoft Ultrasound Gate 416 USB Audio Recorder	Avisoft Bioacoustics, Glienicke/Nordbahn, Germany	https://avisoft.com/
GRAS 46DP-11/8″ LEMO Pressure Standard Microphone Set	Gras Acoustics, Holte, Denmark	https://www.grasacoustics.com/
Stainless steel raised wire bar lid (H-70 mm), with hinged divider – model 1354G	Tecnilab-BMI, Someren, The Netherlands	https://tecnilab-bmi.nl/
Rat tunnels with RSV wire hangers	Carfil Quality, Oud-Turnhout, Belgium	https://www.carfil.eu/en/product/rat-tunnels-a597

### Experimental model and study participant details

All procedures were performed in accordance with the guidelines of the Council of the European Union and authorized by the local Animal Ethics Committee (P122/2022). For both experiments, male and female Sprague-Dawley rats (Charles River Laboratories, France) arrived at our facility shortly after weaning (∼ postnatal day (PND) 24). All rats had no previous experience with other behavioral test procedures. The animals were randomly housed in same-sex groups of 4 in polycarbonate type IV cages (33 × 53 × 19 cm) with high stainless steel wire bar lids (7 cm) allowing rats to exhibit natural rearing behavior even when fully grown. A layer of bedding was provided, along with shredded paper, wooden sticks and a red hanging transparent tube as enrichment. Environmental conditions were controlled (light on: 7 a.m. to 7 p.m., temperature: 21°C–24°C, humidity: 30–40%) and food and water were provided *ad libitum*. All behavioral test procedures were performed in the morning (8 a.m. - 2 p.m.) during the light phase.

### Method details

#### Experiment 1

In experiment 1, we assessed whether playback of USV is emotionally contagious to the receiver. To this end, we developed a new behavioral assay with the potential to assess bidirectional (i.e., negative and positive) emotional contagion in rats via playback, referred to as “the emotional contagion paradigm” from now on. To this aim, both male (*N* = 12) and female (*N* = 12) rats were included, and all rats were tested as juveniles and again as young adults.

#### Juveniles

Shortly after weaning (∼PND 24), rats arrived at our facility. From ∼ PND 25–27, all rats were handled for three consecutive days (5 min per rat per day) according to a standardized protocol to reduce stress during experimental procedures. Subsequently, they were subjected to the emotional contagion paradigm from ∼PND 28–31. Rats were weighed daily during both handling and the emotional contagion paradigm (weight: 108.02 ± 2.81 g (males), 94.59 ± 2.81 g (females)).

#### Adults

While rats were maturing, no procedures were performed on the animals except for regular health checks (e.g., weight measurement) and weekly cage cleaning. At PND ∼78–81, all rats were re-subjected to the emotional contagion paradigm while their weight was monitored daily (weight: 442.29 ± 13.07 g (males), 267.16 ± 7.41 g (females)).

#### The emotional contagion paradigm – Experiment 1

Rats innately produce 22-kHz USV and 50-kHz USV to communicate a negative and positive affective state, respectively. In the emotional contagion paradigm, we assess whether these calls are contagious and can induce a negative (by 22-kHz USV) and a positive (by 50-kHz USV) affective state in the receiver. This is done by combining playback of these calls while measuring the acoustic startle response (ASR). The ASR is a reflex, and the strength of this reflex depends on the affective state of the animal (increased by a negative and decreased by a positive affective state). An increase or decrease in ASR magnitude gives us therefore a direct indication of a shift in the rat’s affective state in the negative or positive direction. Based on the ethological value of both USV types, we expect 22-kHz USV and 50-kHz USV to be negatively and positively emotionally contagious, respectively. This will be reflected in an increased and decreased ASR compared to a socially neutral acoustic control, respectively.

#### Acoustic stimuli used for playback

In total, three different acoustic stimuli were used for playback (i.e., 50-kHz USV (plus background noise, BN+), 22-kHz USV (plus background noise, BN+) and no USV (plus background noise, BN+); Figure 1E for a spectrogram of each stimulus). All acoustic stimuli were played at an amplitude of ∼70 dB, which was measured by the GRAS 46DP-11/8″ LEMO Pressure Standard Microphone Set (Gras Acoustics, Denmark) inside the animal enclosure ∼14 cm in front of the speaker to mimic the animal’s perspective. All files were validated by previous work.^24^^,^^25^^,^^28^ Acoustic stimuli were presented during the ASR test through an UltraSoundGate Player 116 (Avisoft Bioacoustics, Germany) connected to an Ultrasonic Dynamic Speaker Vifa (Avisoft Bioacoustics, Germany) placed ∼4 cm in front of the animal’s enclosure in the startle box (Figure 1C).

##### 50-kHz USV (BN+)

A sequence of 13 natural 50-kHz USV calls was repeated for ∼20 min (i.e., ∼340 repetitions, total played calls: ∼4420, total call time: ∼309 s). Of the 13 calls, 10 were frequency-modulated and 3 were flat, ensuring a high number of frequency-modulated calls in a short time frame. The calls had the following characteristics: call duration 0.07 ± 0.01 s, peak frequency 61.24 ± 1.75 kHz, bandwidth 4.63 ± 1.21 kHz, frequency modulation 31.68 ± 4.62 kHz. The calls were originally produced by an adult male albino rat exposed to a cage that contained scents of a cage mate.^53^ Except for the calls, the audio file also contained background noise (BN+) that the rat produced by exploring the cage (for more information, please see^53^).

##### 22-kHz USV (BN+)

A sequence of 29 natural 22-kHz USV calls was repeated for ∼20 min (i.e., ∼20 repetitions, total played calls: ∼580, total call time: ∼685 s). The calls had the following characteristics: call duration 1.18 ± 0.06 s, peak frequency 23.61 ± 0.07 kHz, bandwidth 1.37 ± 0.05 kHz, frequency modulation 1.90 ± 0.09 kHz. These calls were produced by an adult male albino rat after receiving foot shocks (for more information, please see^54^). Background noise was added through the merge files option in Avisoft SASLabPro to control for acoustic confounds ensuring that acoustic conditions could be directly compared.

##### No USV (BN+)

An audio file with no USV but solely background noise was used as an acoustic control stimulus.

#### Procedure

On all days, the set-up was cleaned in between animals with 70% ethanol and rats were acclimatized to a holding room for ∼30 min prior to testing. A visual depiction of the experimental timeline is provided in Figure 2A.

##### Day 1

Rats were habituated to the acoustic startle equipment. To this end, they were placed for 300 s in the animal enclosure of the acoustic startle box (constant 60 dB white noise present).

##### Day 2–4

On these days, positive, negative or no emotional contagion was induced via playback of 50-kHz USV (BN+), 22-kHz USV (BN+), and no USV (BN+) during the ASR test, respectively. On each day, all rats underwent the same ASR test while simultaneously hearing playback. The ASR test consisted of 300 s of acclimatization (constant 60 dB white noise), after which noise bursts of 7 different intensities (78, 85, 92, 99, 106, 113, and 120 dB) were administered. All intensities were presented 10 times (i.e., 10 blocks with a total of 70 noise bursts) with a random interval between the noise bursts of 5–30 s (total test time: ∼25 min). The presentation order of the noise burst intensities was random, but organized such that each intensity was presented once per block. Based on previous studies,^31^^,^^32^^,^^55^ a wide range of different intensities of noise bursts was presented below and above the ASR threshold to avoid ceiling and floor effects. Playback was initiated after the 5 min acclimatization period and 30 s before the first noise burst was presented, whereafter it was played continuously until the end of the ASR test. The length and amplitude of the playback was based on extensive pilot studies. Three acoustic stimuli sequences were used, with each stimulus presented equally often on the second, third, or fourth testing day across animals to avoid sequence effects. The acoustic stimuli sequences were balanced between the sexes.

#### Response measures

*Startle reactivity*: The ASR was measured using the SR-LAB startle response system (San Diego Instruments, US). Animals were placed in the cylindrical animal enclosure placed in a soundproof isolation chamber (∼33 × 33 × 48 cm). Medium (15.24 cm (L), 5.72 cm (Ø)) and large (20.32 cm (L), 8.89 cm (Ø)) animal enclosures were used for juvenile and adult rats throughout the experiment, respectively. The animal enclosure was fixed on a Plexiglas base (∼20 × 13 cm) with a piezoelectric accelerometer underneath. The accelerometer detected the rat’s startle response for 200 ms after the onset of each noise burst at a frequency of 200 Hz, and further processed by the SR-LAB software and digitized as data points in mV. The maximum startle response recorded was used as response measure throughout the study. Prior to the study, the startle boxes were calibrated using the SR-LAB standardization unit, to ensure similar response sensitivity across boxes.

*Response calls*: During the ASR test, USV emitted by the test rat were recorded using an UltraSoundGate condenser microphone (CM16, Avisoft Bioacoustics, Germany) mounted in the right corner of the startle chamber (∼4 cm away from the rat). The microphone was connected to a computer via an Avisoft Ultrasound Gate 416 USB Audio Device, where the USV were recorded with Avisoft Recorder 4.3.01 (sampling rate: 250,000 Hz; format: 16 bit).

#### Experiment 2

Experiment 2 served two main goals. First of all, we aimed to assess the robustness of the startle attenuation by 50-kHz USV (BN+) playback observed in experiment 1. Second, we aimed to better understand the ethological relevance of 22-kHz USV. In experiment 1, 22-kHz USV playback included background noise from a rat walking through its cage (BN+). However, because 22-kHz USV function as alarm calls and are typically emitted during freezing behavior, the presence of movement-related background noise may reduce their ethological validity. A sequence of 22-kHz USV presented without background noise (BN−) may therefore more closely resemble a natural alarm-calling situation. To test this, experiment 2 used a modified version of the emotional contagion paradigm with different acoustic stimuli in an independent cohort of rats. Both male (*N* = 16) and female (*N* = 16) rats were included, and tested as juveniles and as young adults.

#### Juveniles

As in experiment 1, rats arrived shortly after weaning (∼PND 24) at our facility. From ∼ PND 25–27, all rats were handled for three consecutive days (5 min per rat per day) according to a standardized protocol to reduce stress during experimental procedures. Subsequently, they were subjected to the emotional contagion paradigm from ∼PND 28–32. Rats were weighed daily during both handling and the emotional contagion paradigm (weight: 102.50 ± 2.32 g (males), 97.14 ± 1.40 g (females)).

#### Adults

As in experiment 1, while rats were maturing, no procedures were performed on the animals except for regular health checks (e.g., weight measurement) and weekly cage cleaning. At ∼ PND 78–82, they were re-subjected to the emotional contagion paradigm while their weight was monitored daily (weight: 386.99 ± 7.58 g (males), 245.46 ± 4.92 g (females)).

#### The emotional contagion paradigm – Experiment 2

##### Acoustic stimuli used for playback

In the second experiment, four different acoustic stimuli were used for playback (i.e., 50-kHz USV (BN+), no USV (BN+), 22-kHz USV (BN−), and no USV (BN−)). As in experiment 1, all acoustic stimuli were played at an amplitude of ∼70 dB, which was measured by the GRAS 46DP-11/8″ LEMO Pressure Standard Microphone Set (Gras Acoustics, Denmark) inside the animal enclosure ∼14 cm in front of the speaker to mimic the animal’s perspective. Acoustic stimuli were presented during the ASR test through an UltraSoundGate Player 116 (Avisoft Bioacoustics, Germany) connected to an Ultrasonic Dynamic Speaker Vifa (Avisoft Bioacoustics, Germany) placed ∼4 cm in front of the animal’s enclosure in the startle box (Figure 1C).

##### 50-kHz USV (BN+)

This audio file was identical to the one used in experiment 1.

##### No USV (BN+)

This audio file was identical to the one used in experiment 1.

##### 22-kHz USV (BN−)

As in experiment 1, a sequence of 29 natural 22-kHz USV was repeated for 20 min (see experiment 1 for further details). However, in experiment 2, the 22-kHz USV playback did not contain background noise to achieve more naturalistic conditions (BN−).

##### No USV (BN−)

An additional acoustic control condition where no USV and no background noise (BN−) was included.

#### Procedure

On all days, the set-up was cleaned in between animals with 70% ethanol and rats were acclimatized to a holding room for ∼30 min prior to testing. A visual depiction of the experimental timeline is provided in Figure 2F.

##### Day 1

The habituation was executed identical to experiment 1.

##### Day 2–5

On these days, the induction of positive emotional contagion by 50-kHz USV (BN+) playback, negative emotional contagion by 22-kHz USV (BN-) playback and no emotional contagion by no USV (BN+) and by no USV (BN-) playback was assessed. On each day, rats underwent the ASR test while simultaneously hearing playback. The ASR test was identical to the one used in the first experiment. Playback of 50-kHz USV (BN+), 22-kHz USV (BN-), no USV (BN+), and no USV (BN-) was initiated after the 5 min acclimatization period and 30 s before the first noise burst, whereafter it was played continuously until the end of the ASR test. Four acoustic stimuli sequences were applied, composed so that stimuli were presented evenly across the animals on the second, third, fourth and fifth day of testing to avoid sequence effects. The acoustic stimuli sequences were balanced between the sexes.

#### Response measures

##### Startle reactivity

The ASR was measured as in experiment 1.

### Quantification and statistical analysis

#### Startle reactivity

To determine the minimum noise burst intensity required to elicit a reliable startle response during the acoustic control playback (no USV (BN+)), the data was inspected (Figure S1) revealing a prominent startle response starting from 106 dB noise bursts. Since our study focuses on the modulation of the startle response as a measure of emotional contagion, subsequent analyses were restricted to noise burst intensities of 106 dB, 113 dB, and 120 dB.

To assess whether the induction of emotional contagion (experiment 1 and 2) was successful, the acoustic startle response (ASR) was analyzed as a function of playback. We hypothesized that 50-kHz USV (BN+) playback would attenuate the ASR compared to acoustic control conditions, whereas 22-kHz USV (BN + or BN-) would enhance it. Because the ASR data is strictly positive and bounded, a negative binomial multilevel regression model was fitted unless otherwise stated.

#### Startle reactivity – statistical approach

Analyses were performed separately for juvenile males, juvenile females, adult males, and adult females, for both experiment 1 and 2. In the initial analyses, noise burst intensities were pooled and a model was fitted for each group predicting the startle response by playback condition (3-level factor for experiment 1: 22-kHz USV (BN+), no USV (BN+), 50-kHz USV (BN+); 4-level factor for experiment 2: no USV (BN-), 22-kHz USV (BN-), no USV (BN+), 50-kHz USV (BN+)). This was followed by noise burst intensity-specific analyses for each group where the startle response was predicted by playback condition (3-level factor for experiment 1: 22-kHz USV (BN+), no USV (BN+), 50-kHz USV (BN+); 4-level factor for experiment 2: no USV (BN-), 22-kHz USV (BN-), no USV (BN+), 50-kHz USV (BN+)), intensity (3-level factor: 106 dB, 113 dB, 120 dB), and their interaction effect. We implemented a forward-selection procedure, which starts with the simplest model including only random intercepts. Next, we incrementally increased the number of random slopes when favored by our model selection procedure. For comparisons between nested models, we used likelihood ratio tests to evaluate whether the more complex model provided a significantly better fit. For comparisons between non-nested models, we relied on the comparison of AIC and BIC values. An overview of the best-fitting models is provided in Table S1. In our initial investigation of the emotional contagion paradigm, post-hoc pairwise comparisons utilized the Tukey–Kramer correction, allowing comparison among all conditions while avoiding unnecessary loss of statistical power. More conservative corrections are ideal for replication studies, yet can reduce power, particularly with smaller sample sizes and a larger number of comparisons.^56^^,^^57^

In a next step, sex and age differences in startle modulation by 50-kHz USV (BN+) playback were examined. Importantly, the ASR is produced by the entire body of the rat and transmitted through the hind limbs on the surface, which is in turn measured as startle response magnitude in mV. This measure therefore depends highly on body weight, which differs with age and sex.^35^^,^^36^ To correct for this, raw startle responses were transformed to percentage startle modulation by 50-kHz USV (BN+) compared to no USV (BN+). This percentage was calculated for each rat per noise burst intensity at juvenile and adult ages according to the following formula^30^^,^^58^:

$$\text{Startle}\;\text{modulation}\;\left(\%\right)=\left(\frac{50-\text{kHz}\;\text{USV}\;\left(\text{BN}+\right)-\text{no}\;\text{USV}\;\left(\text{BN}+\right)}{\text{no}\;\text{USV}\;\left(\text{BN}+\right)}\right)\;*\;100$$

This formula provides an intuitive measure in which a negative and positive value indicates an attenuated or enhanced startle response by 50-kHz USV (BN+) relative to no USV (BN+), respectively. More specifically, a percentage of −40% corresponds to a 40% decrease in startle response to 50-kHz USV (BN+) compared to no USV (BN+). To examine sex and age differences, a separate model was built for data from 1) juvenile males and females (sex effect in juveniles), 2) adult males and females (sex effect in adults), 3) juvenile and adult males (age effect in males), and 4) juvenile and adult females (age effect in females). For all groups, a multilevel linear model was used, since percentage startle modulation has no lower bound of 0 (i.e., it can be negative). For all comparisons, percentage startle modulation was predicted by sex (2-level factor: male, female) or age (2-level factor: juvenile, adult) for the pooled analysis, and by the main and interaction effects of sex (2-level factor: male, female) or age (2-level factor: juvenile, adult) and intensity (3-level factor: 106 dB, 113 dB, 120 dB) for the intensity-specific analyses. Further model selection was performed to determine the random effect structure (Table S1). At significance, Tukey-Kramer corrected pairwise comparisons were applied. This was followed by an assessment of intra-individual stability of positive emotional contagion through maturation, by performing a Spearman rank correlation test between percentage startle modulation at juvenile and adult age. Male and female data was pooled, since no sex differences were detected in the previous analyses.

Finally, the temporal dynamics of startle modulation by 50-kHz USV (BN+) playback was assessed, and more specifically its evolution across blocks of playback presentation. The data of males, females, adults and juveniles were pooled since no sex or age effects were detected in previous analyses. A negative binomial multilevel model was fitted to the data with playback (2-level factor: 50-kHz USV (BN+), no USV (BN+)), block number (continuous, range: 1–10), and their interaction as predictors. The random effect structure was determined via model selection (Table S1). Since block number was treated as a continuous variable to ensure model convergence, no *post-hoc* pairwise comparisons of USV playback per block number could be conducted.

#### Response calls

USV emitted by juvenile rats (both males and females) during experiment 1 were analyzed. The analysis focused on USV emitted during the playback period, excluding the 5-min acclimatization phase preceding it. To this end, high-resolution spectrograms (frequency resolution: 0.488 kHz, time resolution: 0.512 ms) were generated by applying a Fast-Fourier transform (512 FFT length, 100% frame, Hamming window, 75% time window overlap) using Avisoft SASLabPro 5.3.01 Software. USV were manually labeled and the software automatically calculated call number, duration (ms), peak frequency (kHz) and frequency modulation (kHz, i.e., the difference between the highest and lowest frequency of the call). Based on previous work,^45^^,^^59^^,^^60^^,^^61^ USV were subdivided into four categories following duration and peak frequency: typical 22-kHz USV (duration *>*300 ms, peak frequency *<*32 kHz), atypical 22-kHz USV (duration ≤300 ms, peak frequency *<*32 kHz), typical 50-kHz USV (duration ≤300 ms, peak frequency ≥32 kHz), or atypical 50-kHz USV (duration *>*300 ms, peak frequency ≥32 kHz). As the majority of calls were typical 50-kHz USV, subsequent analyses focused on this subtype.

#### Response calls – statistical approach

The number of 50-kHz USV was analyzed as a function of USV playback using a negative binomial multilevel model. Model selection was performed to select the best-fitting model (Table S1), followed by Tukey-Kramer corrected *post-hoc* pairwise comparisons. Acoustic features (i.e., duration, peak frequency, and frequency modulation) of the typical 50-kHz USV were compared between USV playback conditions using a one-way ANOVA analysis, or when assumptions were violated, an appropriate non-parametric (i.e., Kruskal-Wallis test) and heteroscedastic (i.e., Welch test) alternative, followed by Bonferroni-corrected *post-hoc* pairwise comparisons upon significance. Previously, we showed a link between caller profile and ASR responsivity.^45^ Consistent with this classification, rats were classified based on their call rate during 50-kHz USV (BN+) playback into high callers (>4 typical 50-kHz USV) and low callers (≤4 typical 50-kHz USV). To compare the effect of ASR modulation by playback between high callers and low callers, a separate negative binomial multilevel model was fitted for both groups. Startle response was predicted by playback (3-level factor: 22-kHz USV (BN+), no USV (BN+), 50-kHz USV (BN+)) for the pooled analysis, and by playback (3-level factor: 22-kHz USV (BN+), no USV (BN+), 50-kHz USV (BN+)), intensity (3-level factor: 106 dB, 113 dB, 120 dB), and their interaction for the intensity-specific analyses. Model selection identified a best-fitting models for each analysis (Table S1), followed by Tukey-Kramer corrected *post-hoc* pairwise comparisons.

All statistical analyses were performed in Rstudio (R core team, 2021; version: 4.1.2) using glmmTMB, lme4, emmeans and lmerTest. Data figures were created in Rstudio (R core team, 2021; version: 4.1.2) and compiled using GraphPad Prism (version 10). Explanatory illustrations (e.g., Figure 1A) were made using BioRender (BioRender.com), ChatGPT (GPT-5.6 Luna) or PowerPoint (version 2608). Data is represented as mean ± SE. There was no missing data. No major over/underdispersion was detected in all negative binomial models (disp. ratio = 0.86–1.14). Some models displayed multicollinearity (VIF≤17.06), which could be attributed to the interaction between playback and intensity. Removing the interaction term resolved the multicollinearity (VIF≤1.56), while maintaining the significant main effects of both playback (all *χ*^2^ ≥ 12.98, *p* ≤0 .002) and intensity (all *χ*^2^ ≥ 84.72, *p* ≤ 0 .0001) indicating the robustness of our key results.
