# Supplementary material for: Positive emotional contagion by laughter-like 50-kHz ultrasonic vocalizations in rats
Source: iScience. 2026 Nov 2;29(11):118087. doi: 10.1016/j.isci.2026.118087 (PMC13756053; doi:10.1016/j.isci.2026.118087)
Supplement: Supplementary file 1 [file mmc1.pdf]

iScience, Volume *29*

Supplemental information

Positive emotional contagion by laughter-like

50-kHz ultrasonic vocalizations in rats

Emilie Bartsoen, Robin Vloeberghs, Parnian Saberi, Kobe Desender, and Markus Wöhr

Supplemental information

Supplemental figures

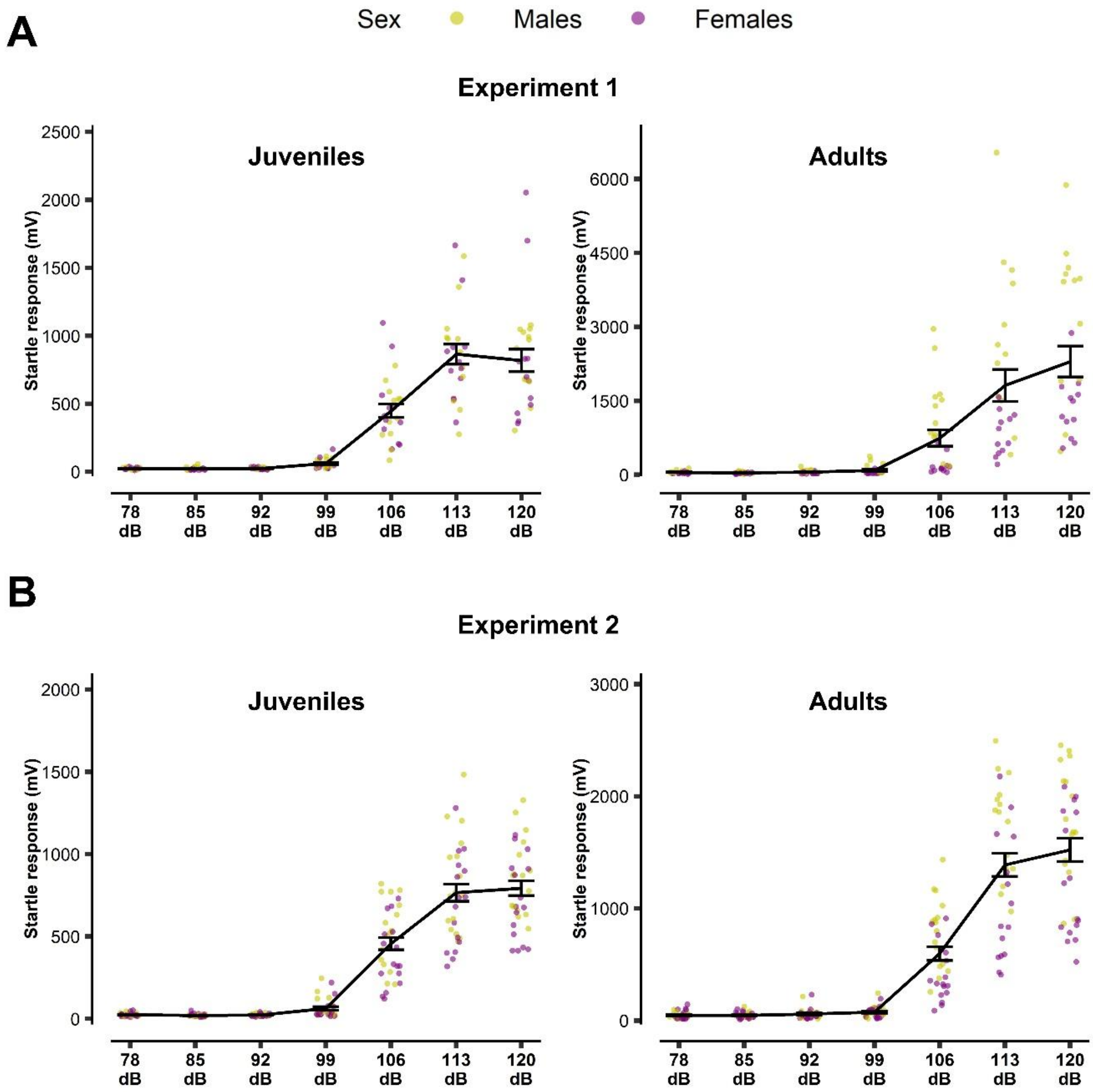

Figure S1. The acoustic startle response (ASR) to all noise burst intensities during playback of no USV (BN+) in experiment 1 and 2, related to STAR Methods. Line graphs showing the ASR to different noise burst intensities in juvenile and adult rats tested in experiment 1 (N = 24) (A) and experiment 2 (N = 32) (B) measured during playback of no USV (BN+). Please note that a robust startle response was observed in response to the three loudest noise burst intensities (i.e., 106 dB, 113 dB, 120 dB). The mean±SE is plotted per noise burst intensity. Each dot represents a rat.

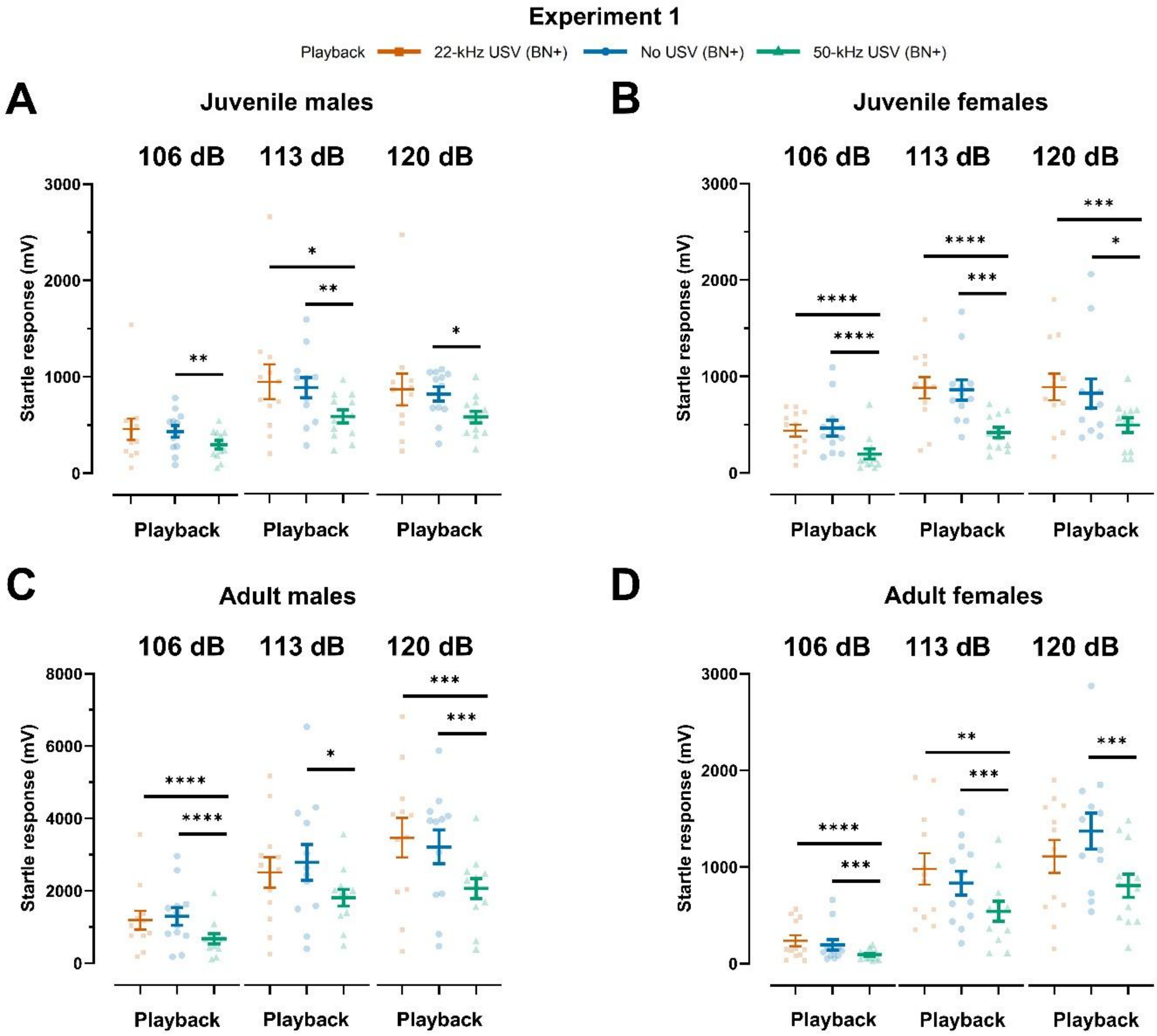

Figure S2. Post-hoc comparisons of the startle reactivity between playback conditions per noise burst intensity in experiment 1, related to Figure 2. Graphs depict the results of *post-hoc* comparisons for experiment 1. Using Tukey-Kramer correction, startle reactivity was compared between playback conditions per noise burst intensity. Mean±SE is plotted. Each dot represents a rat (N = 12 per age and sex group). **p*<.05, ***p*<.01, ****p*<.001, *****p*<.0001.

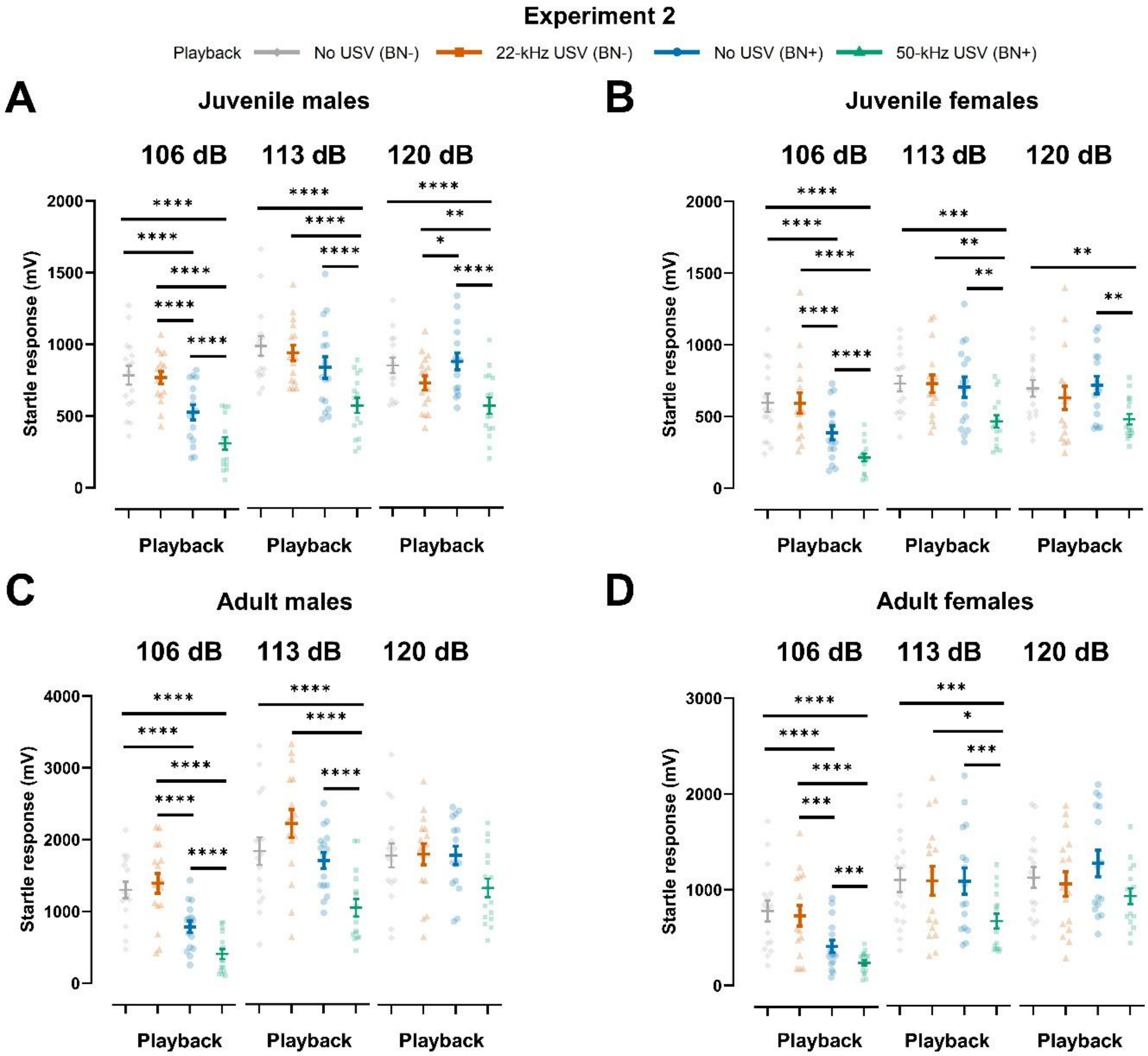

Figure S3. Post-hoc comparisons of the startle reactivity between playback conditions per noise burst intensity in experiment 2, related to Figure 2. Graphs depict the results of *post-hoc* comparisons for experiment 2. Using Tukey-Kramer correction, startle reactivity was compared between playback conditions per noise burst intensity. Mean±SE is plotted. Each dot represents a rat (N = 16 per age and sex group). **p*<.05, ***p*<.01, ****p*<.001, *****p*<.0001.

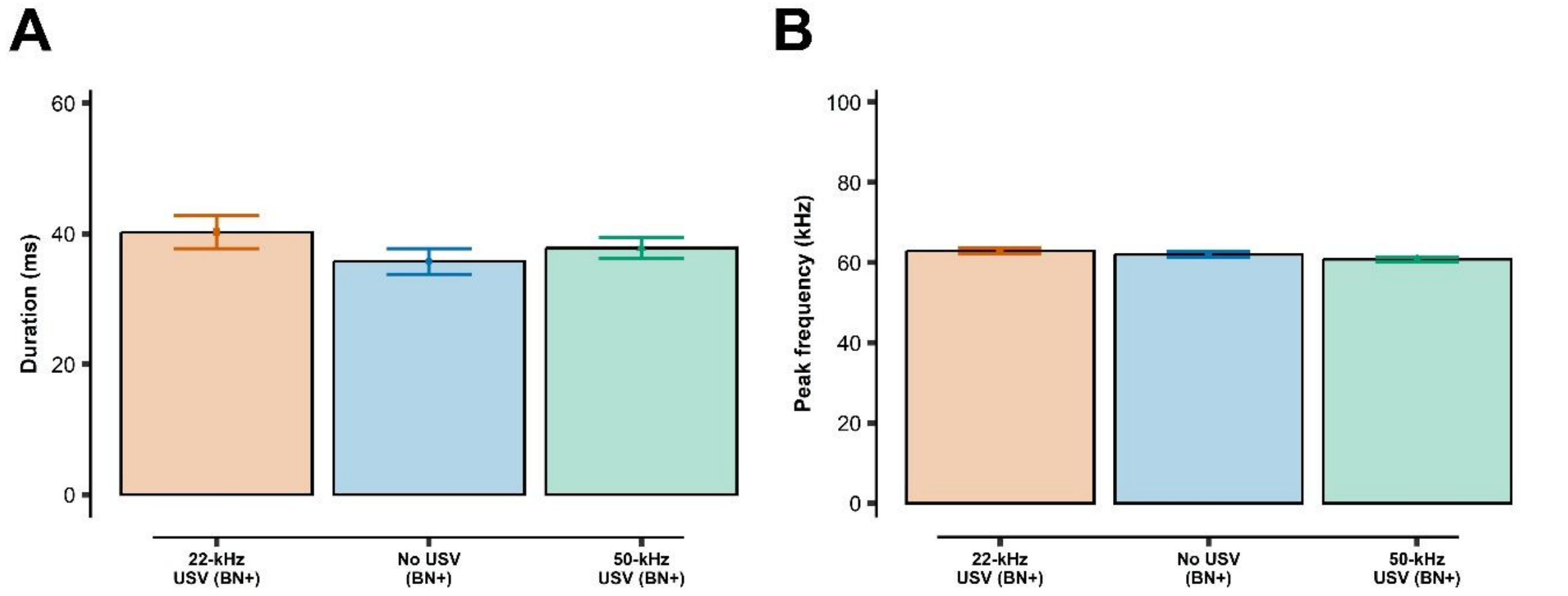

Figure S4. Duration and peak frequency of typical 50-kHz USV in each playback condition, related to Figure 3. Bar plots (mean±SE) depict duration (ms) and peak frequency (kHz) between playback conditions of typical 50-kHz USV (analyzed on call level, N = 1205). No differences were found.

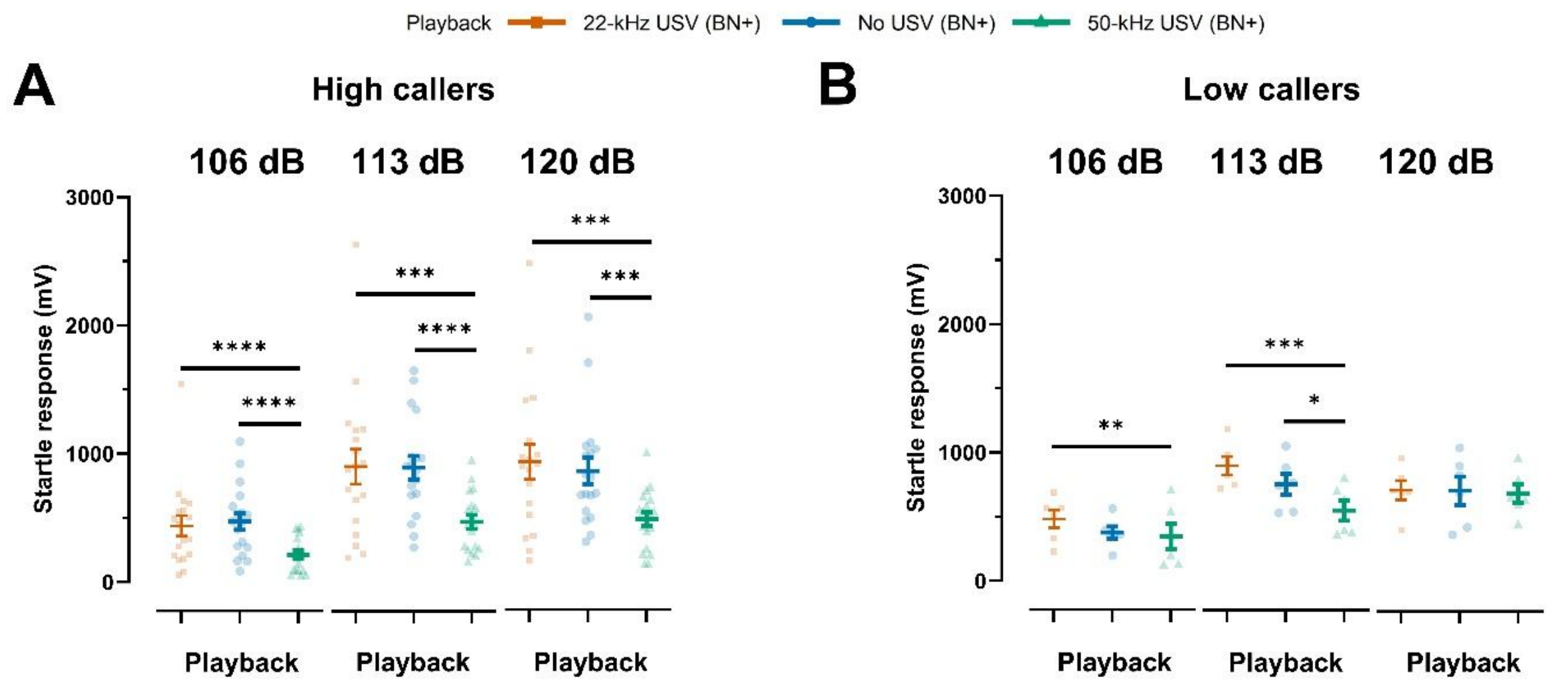

Figure S5. Post-hoc comparisons of the startle reactivity between playback conditions per noise burst intensity – high versus low callers, related to Figure 3. Graphs depict the results of *post-hoc* comparisons between high (N = 18) vs. low (N = 6) callers. For both groups, startle reactivity was compared between playback conditions per noise burst intensity using Tukey-Kramer correction. Mean±SE is plotted. Each dot represents a rat. **p*<.05, ***p*<.01, ****p*<.001, *****p*<.0001.

Supplemental tables

Table S1. An overview of the best fitting model per statistical comparison, related to STAR Methods.

	**Startle modulation by playback – Experiment 1**
**Pooled intensity analyses**	
**Juvenile males**	$\text{ASR} = \text{playback} + (1 + \text{playback} \mid \text{subject})$
**Juvenile females**	$\text{ASR} = \text{playback} + (1 + \text{playback} \mid \text{subject})$
**Adult males**	$\text{ASR} = \text{playback} + (1 \mid \text{subject})$
**Adult females**	$\text{ASR} = \text{playback} + (1 + \text{playback} \mid \text{subject})$
**Intensity-specific analyses**	
**Juvenile males**	$\text{ASR} = \text{playback} * \text{intensity} + (1 + \text{playback} \mid \text{subject})$
**Juvenile females**	$\text{ASR} = \text{playback} * \text{intensity} + (1 + \text{playback} \mid \text{subject})$
**Adult males**	$\text{ASR} = \text{playback} * \text{intensity} + (1 + \text{playback} \mid \text{subject})$
**Adult females**	$\text{ASR} = \text{playback} * \text{intensity} + (1 + \text{playback} + \text{intensity} \mid \text{subject})$
	**Startle modulation by playback – Experiment 2**
**Pooled intensity analyses**	
**Juvenile males**	$\text{ASR} = \text{playback} + (1 \mid \text{subject})$
**Juvenile females**	$\text{ASR} = \text{playback} + (1 + \text{playback} \mid \text{subject})$
**Adult males**	$\text{ASR} = \text{playback} + (1 + \text{playback} \mid \text{subject})$
**Adult females**	$\text{ASR} = \text{playback} + (1 + \text{playback} \mid \text{subject})$
**Intensity-specific analyses**	
**Juvenile males**	$\text{ASR} = \text{playback} * \text{intensity} + (1 + \text{playback} \mid \text{subject})$
**Juvenile females**	$\text{ASR} = \text{playback} * \text{intensity} + (1 + \text{playback} \mid \text{subject})$
**Adult males**	$\text{ASR} = \text{playback} * \text{intensity} + (1 + \text{playback} \mid \text{subject})$
**Adult females**	$\text{ASR} = \text{playback} * \text{intensity} + (1 + \text{playback} \mid \text{subject})$
	**Startle modulation by playback – High vs. low callers**
**Pooled intensity analyses**	
**High callers**	$\text{ASR} = \text{playback} + (1 + \text{playback} \mid \text{subject})$
**Low callers**	$\text{ASR} = \text{playback} + (1 \mid \text{subject})$
**Intensity-specific analyses**	
**High callers**	$\text{ASR} = \text{playback} * \text{intensity} + (1 + \text{playback} \mid \text{subject})$
**Low callers**	$\text{ASR} = \text{playback} * \text{intensity} + (1 \mid \text{subject})$
	**Sex effects**
**Pooled intensity analyses**	
**Juveniles**	$\text{Startle modulation (\%)} = \text{sex} + (1 + \text{sex} \mid \text{subject})$
**Adults**	$\text{Startle modulation (\%)} = \text{sex} + (1 \mid \text{subject})$
**Intensity-specific analyses**	
**Juveniles**	$\text{Startle modulation (\%)} = \text{sex} * \text{intensity} + (1 + \text{sex} \mid \text{subject})$
**Adults**	$\text{Startle modulation (\%)} = \text{sex} * \text{intensity} + (1 \mid \text{subject})$
	**Age effects**
**Pooled intensity analyses**	
**Males**	$\text{Startle modulation (\%)} = \text{age} + (1 \mid \text{subject})$
**Females**	$\text{Startle modulation (\%)} = \text{age} + (1 + \text{age} \mid \text{subject})$
**Intensity-specific analyses**	
**Males**	$\text{Startle modulation (\%)} = \text{age} * \text{intensity} + (1 + \text{age} \mid \text{subject})$
**Females**	$\text{Startle modulation (\%)} = \text{age} * \text{intensity} + (1 + \text{age} \mid \text{subject})$
	**Temporal dynamics of startle modulation by playback**
	$\text{ASR} = \text{playback} * \text{block number} + (1 + \text{playback} + \text{block number} \mid \text{subject})$

A forward-selection procedure was used. This started with the simplest model including only random intercepts and incrementally increased the number of random slopes when favored by our model selection procedure. For comparisons between nested models, we used likelihood ratio tests to evaluate whether the more complex model provided a significantly better fit. For comparisons between non-nested models, we relied on the comparison of AIC and BIC values.

Table S2. The observed percentages of each USV type per playback condition, related to Figure 3.

	22-kHz USV (BN+)	No USV (BN+)	50-kHz USV (BN+)
**Typical 50-kHz USV**	98.58 %	99.08 %	94.51 %
**Typical 22-kHz USV**	0.00 %	0.00 %	0.78 %
**Atypical 50-kHz USV**	0.35 %	0.61 %	1.41 %
**Atypical 22-kHz USV**	1.06 %	0.31 %	3.29 %

Supplemental Text

Text S1. Noise burst intensity-specific results of experiment 2, related to Figure 2.

Further analyses at the level of individual noise burst intensities revealed a clear ASR modulation depending on the acoustic stimulus presented (main effect playback: $\chi^2(2)=105.69$, $p<.0001$ (juvenile males), $\chi^2(2)=42.72$, $p<.0001$ (juvenile females), $\chi^2(2)=57.54$, $p<.0001$ (adult males), $\chi^2(2)=47.19$, $p<.0001$ (adult females)). The ASR magnitude increased with increasing noise burst intensity (main effect intensity: $\chi^2(2)=127.43$, $p<.0001$ (juvenile males), $\chi^2(2)=162.13$, $p<.0001$ (juvenile females), $\chi^2(2)=283.05$, $p<.0001$ (adult males), $\chi^2(2)=306.28$, $p<.0001$ (adult females)), but the shape and steepness of this increase differed clearly between playback conditions (interaction effect: $\chi^2(6)=87.69$, $p<.0001$ (juvenile males), $\chi^2(6)=88.96$, $p<.0001$ (juvenile females), $\chi^2(6)=115.67$, $p<.0001$ (adult males), $\chi^2(6)=121.61$, $p<.0001$ (adult females)). Specifically, 50-kHz USV (BN+) induced a marked attenuation of ASR across all four age and sex groups, with the strongest effects observed at the two lowest noise burst intensities. In contrast, no USV (BN+) playback only modestly reduced ASR magnitude compared to no USV (BN-). Importantly, this effect was restricted to the lowest noise burst intensity and was substantially smaller than the attenuation induced by 50-kHz USV (BN+), indicating that background noise (BN+) alone cannot account for the robust ASR suppression across noise burst intensities during 50-kHz USV presentation. Finally, 22-kHz USV (BN-) did not potentiate the ASR, as its magnitude was not different from no USV (BN-) (Figure S3 for *post-hoc* results).
